# Peristaltic transport of Sutterby nanofluid flow in an inclined tapered channel with an artificial neural network model and biomedical engineering application

**DOI:** 10.1038/s41598-023-49480-9

**Published:** 2024-01-04

**Authors:** P. Chinnasamy, R. Sivajothi, S. Sathish, Mohamed Abbas, V. Jeyakrishnan, Rajat Goel, Mohammed S. Alqahtani, K. Loganathan

**Affiliations:** 1 Department of Computer Science and Engineering, MLR Institute of Technology, Hyderabad, Telangana India; 2 Department of Management, R L Institute of Management Studies (A Unit of Subbalakshmi Lakshmipathy College of Science), Madurai, Tamil Nadu India; 3 Department of Mathematics, School of Science, National Institute of Technology, Tadepalligudem, Andhra Pradesh India; 4 Electrical Engineering Department, College of Engineering, King Khalid University, 61421 Abha, Saudi Arabia; 5 Department of Computer Science and Engineering, Manipal University Jaipur, Jaipur, Rajasthan 303007 India; 6 Radiological Sciences Department, College of Applied Medical Sciences, King Khalid University, 61421 Abha, Saudi Arabia; 7 BioImaging Unit, Space Research Centre, Michael Atiyah Building, University of Leicester, Leicester, LE1 7RH UK; 8 Department of Mathematics and Statistics, Manipal University Jaipur, Jaipur, Rajasthan 303007 India

**Keywords:** Biophysics, Mathematics and computing, Physics

## Abstract

Modern energy systems are finding new applications for magnetohydrodynamic rheological bio-inspired pumping systems. The incorporation of the electrically conductive qualities of flowing liquids into the biological geometries, rheological behavior, and propulsion processes of these systems was a significant effort. Additional enhancements to transport properties are possible with the use of nanofluids. Due to their several applications in physiology and industry, including urine dynamics, chyme migration in the gastrointestinal system, and the hemodynamics of tiny blood arteries. Peristaltic processes also move spermatozoa in the human reproductive system and embryos in the uterus. The present research examines heat transport in a two-dimensional deformable channel containing magnetic viscoelastic nanofluids by considering all of these factors concurrently, which is vulnerable to peristaltic waves and hall current under ion slip and other situations. Nanofluid rheology makes use of the Sutterby fluid model, while nanoscale effects are modeled using the Buongiorno model. The current study introduces an innovative numerical computing solver utilizing a Multilayer Perceptron feed-forward back-propagation artificial neural network (ANN) with the Levenberg–Marquardt algorithm. Data were collected for testing, certifying, and training the ANN model. In order to make the dimensional PDEs dimensionless, the non-similar variables are employed and calculated by the Homotopy perturbation technique. The effects of developing parameters such as Sutterby fluid parameter, Froude number, thermophoresis, ion-slip parameter, Brownian motion, radiation, Eckert number, and Hall parameter on velocity, temperature, and concentration are demonstrated. The machine learning model chooses data, builds and trains a network, and subsequently assesses its performance using the mean square error metric. Current results declare that the improving Reynolds number tends to increase the pressure rise. Improving the Hall parameter is shown to result in a decrease in velocity. When raising a fluid's parameter, the temperature profile rises.

## Introduction

In the past few years, there has been a lot of attention on physico-mathematical and computer simulations of non-Newtonian fluids. Chemicals (including plastics, paints, and polymers), medicines, industrial lubricants, gels, grease, and culinary products like ketchup, yogurt, and honey are all examples of non-Newtonian fluids. Non-Newtonian behavior is also seen in biological systems that cope with oil spills, mudflows, pollution discharge, and highly concentrated sediments. The standard Navier–Stokes equations, which were developed for viscous models, do not well describe non-Newtonian fluids because of their basic characteristics. Several fluid phenomena cannot be reproduced using Newtonian fluid dynamics, such as Weissenberg effects, stress variations, shear-thinning/shear-thickening, elongation, yield stress, relaxation, retardation microstructure, spurt, re-coiling, and fading memory^1–3^. A diversity of rheological concepts have been presented by researchers, such as the Maxwell concept^4^, rheological models, Burgers’ viscoelastic model^5^, Walters-B model^6^, Williamson fluid, second-grade fluid model^7^, Carreau fluid, Oldroyd-B model, Johnson–Segalman fluid, Sisko model^8^, Jeffery model^9^, and FENE-P fluid, to subdue these issues in the Navier–Stokes equations. Maqbool et al.^10^ used a fractional Burger's viscoelastic fluid model to discuss the heat transfer of electrically-conducting fluid over a porous rotating plate. Using rheological working fluids, MHD spinning energy producers apply these types of problems to fluid dynamic processes. Vasu et al.^11^ performed a numerical analysis of the effects of erratic and fluctuating surface fluxes on the distribution of gyrotactic biological convection flow via a stretched sheet by MHD-Casson nanofluid. Shanmugapriya et al.^12^ studied the entropy generation on MHD Carreau liquid over a moving wedge with the influence of thermal radiation. Bioconvection nanofluid flow of shear thinning (tangent hyperbolic) rheological model containing gyrotactic microorganisms across a porous stretched surface was studied by Jakeer and Reddy^13^, who employed the Homotopy perturbation approach. Bhaumik et al.^14^ conducted a study on a physics-aided deep learning model for the prediction of viscosity in nanofluids. This model integrates data-driven models with a physics-based theoretical model. Hayat et al.^15^ examine how the elasticity of the flexible walls affects the peristaltic motion of a power-law fluid. Hayat and Javed^16^ analyze the asymmetric peristaltic flow of a non-Newtonian fluid in an asymmetric channel, taking into account the impacts of compliant wall features. Shahzadi and Nadeem^17^ examined how an angled magnetic field and metallic nanoparticles affected the peristaltic motion of a nanofluid in an annulus subject to convective boundary conditions. Ijaz et al.^18^ explored the use of peristaltic micro-pumps in pharmacological engineering, employing magnetic field control. These micro-pumps were integrated into a non-Newtonian fluid and functioned within a space with partial permeability, enclosed by flexible walls.

A unique mechanism that highly controls the movement of the biophysical fluids (blood, food bolus, urine and chyme) in the human system is known as peristalsis. It is observed that the fluid in a duct is transported without any external pump flow (Tiny blood artery vasomotion, kidney to bladder urine flow, and oesophageal to gastric flow of food are examples of vasomotion); hence this mechanism is remarkable^19–24^. The peristaltic phenomenon is a wavelike manner that happens in the human system through smooth muscle tissues, continuous relaxation, and reduction. Because of their importance in a wide range of physiological and industrial uses, such as bile in the bile duct, the transport of cilia, and nanoscale vasomotion of blood vessels, peristaltic processes have received an extensive study from researchers. Pozrikidis^25^ explored the two-dimensional peristaltic flow in 1987 by taking creeping movements into account and framing the issue using the boundary integral Stokes flow approach. By taking into consideration the Reiner-Philippoff (RPh) fluid model, peristaltically flowing nanofluids via a non-uniform channel were studied by Tahir et al.^26^ for their pseudoplastic and dilatant behaviors. Their findings showed that the nanofluid's energy transfer rate rapidly increased with increasing nanoparticle concentration in the base fluid. Abbasi et al.^27^ explored the irreversibility impacts on MHD peristaltic nanofluid flow via an asymmetric channel with non-uniformity by considering its rheological characteristics. Rafi et al.^28^ studied the analysis of MHD electroosmotic peristaltic flow of Jeffrey nanofluids over a tapered microfluidic asymmetric channel along with the chemical reaction. An incompressible Williamson nanofluid was studied by Bhaumik et al.^29^ during its peristaltic transit in an asymmetrically inclined annular tube, taking into account the effects of a magnetic field, thermophoresis, and Brownian force. Hayat et al.^30^ investigated the effect that compliant walls having on the flow of Sutterby fluid that was peristaltically produced via a vertical conduit. A magnetic field with a consistent intensity is applied in the transverse direction to the flow. Shahzadi and Nadeem^31^ investigated the magnetohydrodynamic peristaltic flow of nanofluid using copper and silver nanoparticles passing through eccentric annuli as the nanoparticles and blood as the base fluid. Riaz et al.^32^ conducted research on the peristaltic flow scheme for a Newtonian fluid inside of a three-dimensional enclosed curved duct that had a rectangular cross-section.

Peristaltic transport is a mechanism characterized by the rhythmic contraction and relaxation of muscles or mechanical pumps, leading to the propagation of fluid through a tube or duct in a wave-like manner. This phenomenon is widely observed in biological systems, such as the movement of food bolus in the digestive tract, urine flow in the urinary system, and blood circulation in small blood vessels. Additionally, peristaltic processes play a vital role in the movement of spermatozoa in the human reproductive system and embryos in the uterus. The applications of peristaltic transport, when combined with artificial neural network (ANN) models, have seen significant advancements in recent years. ANN models have been employed to simulate and predict fluid flow behavior, especially in scenarios involving peristalsis. By using ANN-based simulations, researchers can gain insights into complex fluid dynamics and understand the effects of various parameters on the flow characteristics. In biomedical engineering, ANN models have been integrated with peristaltic transport to design and optimize drug delivery systems. ANN simulations can predict drug dispersion patterns, optimize dosing schedules, and improve the accuracy of drug delivery devices. Furthermore, ANN models have been utilized in microfluidics to control and manipulate fluid flow within microchannels, facilitating precise dosing and mixing for medical diagnostics and lab-on-a-chip applications^33,34^. Environmental engineering has also benefited from ANN-assisted peristaltic transport studies. ANN models are used to predict the movement of sewage, slurry, and industrial effluents in waste management systems, aiding in the design of efficient transport and treatment processes. Additionally, ANN-based simulations have been applied in pollution control efforts to predict pollutant dispersion and develop strategies for remediation. In pharmaceutical manufacturing, ANN models integrated with peristaltic transport have been employed to optimize the handling of sensitive fluids, such as drug formulations, and improve the precision of drug delivery systems. ANN simulations have been instrumental in drug formulation studies, enabling researchers to design pharmaceutical compounds with enhanced stability and efficacy. Food processing industries have also utilized ANN models in conjunction with peristaltic transport to optimize the handling of viscous food products. By accurately predicting fluid flow patterns, ANN simulations enable precise control of mixing and dispensing processes, ensuring consistent product quality. In various industrial processes, ANN-assisted peristaltic transport simulations have been utilized to optimize fluid flow in manufacturing and processing systems. These simulations provide valuable insights into flow control, helping industries achieve more efficient and cost-effective operations. Hayat et al.^35^ explored the peristaltic flow of Sutterby fluid in a planar symmetric channel with electrically conducting fluid being considered by the use of an applied magnetic field. The impact of Hall on the peristaltic motion of Johnson-Segalman fluid in a heated, elastically walled channel was studied by Javed^36^. Zeeshan et al.^37^ investigated the electroosmosis-modulated bio-flow of nanofluid via a rectangular peristaltic pump caused by a complicated moving wave with zeta potential and heat source.

Although a Lorentzian retarding force is often produced by a transverse static magnetic field during the processing of magnetohydrodynamic materials, various additional phenomena are possible. These phenomena include alternating magnetic fields, Alfven waves, magnetic induction, magnetic leakage, magnetic dipoles, ion slip currents, and Hall currents^38^. Hall current and ion slip current may become significant at stronger magnetic fields. The latter increases the regime's electrical conductivity, whereas the former decreases the medium's ability to induce a secondary (cross) flow. The latter deals with the phenomenon of partial ionization, which lowers the electrical conductivity of liquids and gases. Numerous researchers have investigated the effects of ion slip current and Hall current on the intensity and direction of the current density, which in turn changes the impact of the magnetic body force in the context of engineering MHD flows. Shamshuddin et al.^39^ studied exponentially stretched sheet features on the magnetic fluid flow by means of power-law slip velocity, Joule heating, viscous dissipation, and Hall current. Das et al.^40^ conducted an investigation into the peristaltic transport of a copper–water nanofluid within an asymmetric channel, considering the impact of a strong magnetic field. Hayat et al.^41^ investigate the Hall and ion slip effects of mixed convective peristaltic flow of Jeffrey nanofluid in a channel in the presence of viscous dissipation, thermal radiation and joule heating. Dolui et al.^23^ examined the effects of heat radiation and an induced magnetic field on a two-dimensional blood flow via an inclined catheterized artery with numerous stenoses. Bhaumik et al.^42^ a machine learning technique and physics-based relations to investigate the impact of thermal conductivity of water-based nanofluids (Al_2_O_3_, CuO, and TiO_2_). Shahzadi et al.^43^ investigated the impact of electroosmotic forces in an oblique stenosed aneurysmal artery using a fractional second-grade fluid model with ternary nano-particles.

The objective of this study is to demonstrate the feasibility of a mathematical model for the magneto bio-Sutterby blood nanofluid. This will be achieved by conducting simulations to analyze the peristaltic flow of the nanofluid in a tapered inclined channel, considering the influence of hall-current and ion-slip enhancement. In order to modify the flow of magneto bio-Sutterby blood nanofluid caused by peristalsis, it is essential to consider the presence of a tapered channel and the influence of hall current on a non-Newtonian nanofluid. This model possesses various applications in the fields of physiology and industry, encompassing urine dynamics, chyme migration within the gastrointestinal system, and the hemodynamics of small blood arteries. Peristaltic processes are employed for the transportation of spermatozoa within the human reproductive system, as well as for the movement of embryos within the uterus. This paper presents a novel numerical computing solution utilizing MLP feed-forward back-propagation ANN and the Levenberg–Marquardt algorithm. Rigorous data collection ensures thorough testing, certification, and training of the model for optimal performance. The utilization of the Homotopy Perturbation Method (HPM) serves as a problem-solving tool for converting dimensional partial differential equations into dimensionless forms by employing non-similar variables. This study demonstrates the impact of various emerging factors, including the Sutterby fluid parameter, Froude number, thermophoresis, ion-slip parameter, Brownian motion, radiation, Eckert number, and Hall parameter, on the velocity, temperature, and concentration parameters.

## Mathematical formulation

**Figure 1:**
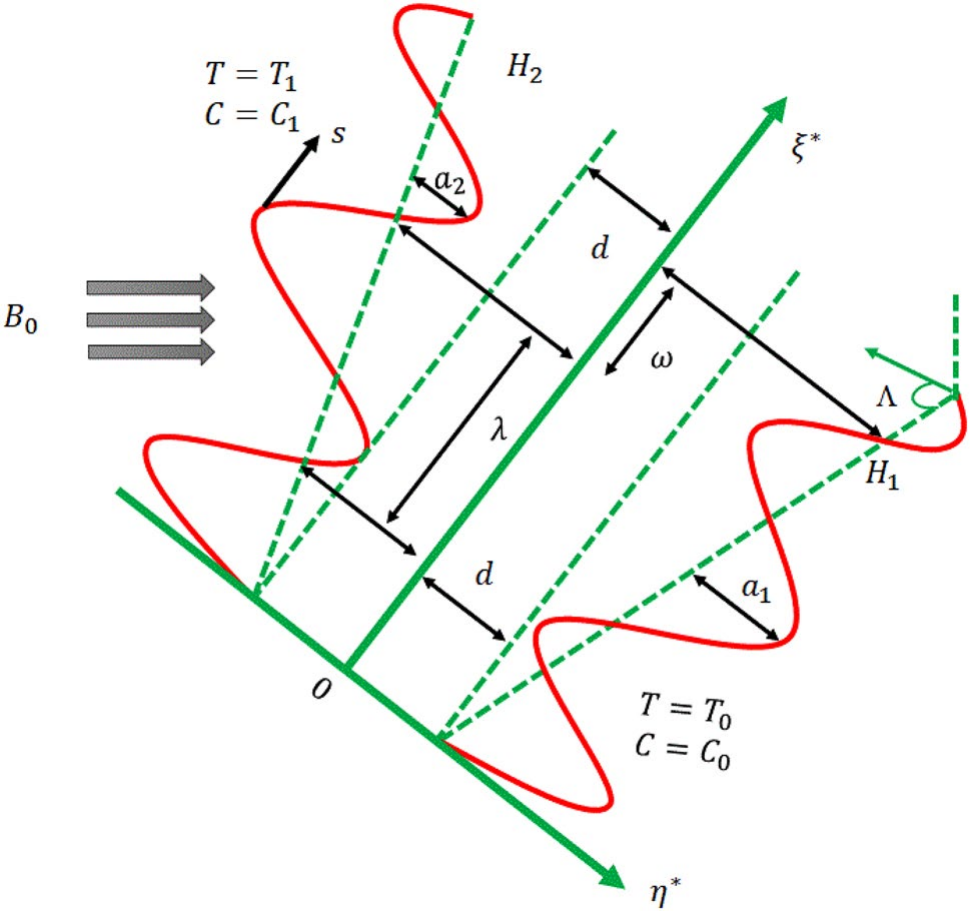
The configuration of the flow.

The current model addresses the peristaltic transport of Sutterby nanofluid under hydromagnetic conditions in an inclined tapered channel, as illustrated in Fig. 1. A two- dimensional Cartesian coordinate system $\left( {\xi^{ * } ,\eta^{ * } } \right)$ is chosen for this modelling where $\xi^{ * }$ and $\eta^{ * }$ are oriented in the Sutterby-nanofluid flow direction (middle of the channel) and perpendicular to the flow path, accordingly.$B_{0}$ is the strength of an uniform magnetic field that is used to implement the flow of nanofluids in their normal direction. Consider the electrically conducting incompressible Sutterby nanofluid. The flow equation takes into consideration the body force. Therefore, the model incorporates Hall and ion-slip currents, accounting for Joule heating and viscous dissipation. Additionally, thermophoretic and Brownian motion phenomena are considered. Using wave trains with different amplitudes and phases allows us to create channel asymmetry.

In a wavy channel, the walls on each side are $\eta^{ * } = h_{1} \,\,$ is the left ${\text{and}}\,\eta^{ * } = h_{2}$ is the right walls of the channel, consequently.

1 $$\left. {h_{2} \left( {\xi^{ * } ,t^{ * } } \right) = d + a_{2} \left( {\frac{2\pi }{\lambda }\left( {\xi^{ * } - st^{ * } } \right)} \right) + m^{ * } \xi^{ * } } \right\}{\text{Left}}\,{\text{wall}}$$

2 $$\left. {h_{1} \left( {\xi^{ * } ,t^{ * } } \right) = - d - a_{1} \left( {\frac{2\pi }{\lambda }\left( {\xi^{ * } - st^{ * } } \right) + \omega } \right) - m^{ * } \xi^{ * } } \right\}{\text{Right}}\,{\text{wall}}$$

where $a_{1}$, $a_{2}$,$s$,$d$,$m^{ * }$,$\omega$ and $\lambda$ represent the following parameters are right wall amplitude, left wall amplitude, phase speed of the wave, channel half width, non-uniform parameter, length of a wave and the phase difference between two waves. A phenomenon known as the phase difference, shown as $\left( {0 \le \omega \le \pi } \right)$, exhibits variation. Additionally, the channel undergoes a transition from an asymmetric state to a symmetric state when the value of w is equal to zero.

Taking into account the aforementioned elements, we get the dimensional form of the equations governing fluid transport^44–46^

3 $$\frac{{\partial u^{*} }}{{\partial \xi^{*} }} + \frac{{\partial v^{*} }}{{\partial \eta^{*} }} = 0$$

4 $$\begin{aligned} & \rho_{f} \left( {\frac{{\partial u^{*} }}{{\partial t^{*} }} + u^{*} \frac{{\partial u^{*} }}{{\partial \xi^{*} }} + v^{*} \frac{{\partial u^{*} }}{{\partial \eta^{*} }}} \right) = - \frac{{\partial p^{*} }}{{\partial \xi^{*} }} + \frac{{\partial \tau_{{\xi^{*} \xi^{*} }}^{*} }}{{\partial \xi^{*} }} + \frac{{\partial \tau_{{\xi^{*} \eta^{*} }}^{*} }}{{\partial \eta^{*} }} + \rho_{f} g\sin \Lambda + \left( {\rho_{p} - \rho_{f} } \right)g\beta \left( {C - C_{0} } \right) \\ & \quad + \frac{{\sigma d_{1}^{2} B_{0}^{2} }}{{\left[ {\left( {1 + \beta_{e} \beta_{i} } \right)^{2} + \beta_{e}^{2} } \right]}}\left( {\beta_{e} v^{*} - \left( {1 + \beta_{e} \beta_{i} } \right)u^{*} } \right) + \left( {1 - C_{0} } \right)g\rho_{f} \alpha \left( {T - T_{0} } \right) \\ \end{aligned}$$

5 $$\begin{aligned} & \rho_{f} \left( {\frac{{\partial v^{*} }}{{\partial t^{*} }} + u^{*} \frac{{\partial v^{*} }}{{\partial \xi^{*} }} + v^{*} \frac{{\partial v^{*} }}{{\partial \eta^{*} }}} \right) = - \frac{{\partial p^{*} }}{{\partial \eta^{*} }} + \frac{{\partial \tau_{{\xi^{*} \eta^{*} }}^{*} }}{{\partial \xi^{*} }} + \frac{{\partial \tau_{{\eta^{*} \eta^{*} }}^{*} }}{{\partial \eta^{*} }} - \rho_{f} g\cos \Lambda \\ & \quad - \frac{{\sigma d_{1}^{2} B_{0}^{2} }}{{\left[ {\left( {1 + \beta_{e} \beta_{i} } \right)^{2} + \beta_{e}^{2} } \right]}}\left( {\beta_{e} u^{*} + \left( {1 + \beta_{e} \beta_{i} } \right)v^{*} } \right) \\ \end{aligned}$$

6 $$\begin{aligned} & \left( {\rho C_{p} } \right)_{f} \left( {\frac{\partial T}{{\partial t^{*} }} + u^{*} \frac{\partial T}{{\partial \xi^{*} }} + v^{*} \frac{\partial T}{{\partial \eta^{*} }}} \right) = k_{f} \left( {\frac{{\partial^{2} T}}{{\partial \xi^{*2} }} + \frac{{\partial^{2} T}}{{\partial \eta^{*2} }}} \right) + \tau_{{\xi^{*} \xi^{*} }}^{*} \frac{{\partial u^{*} }}{{\partial \xi^{*} }} + \tau_{{\eta^{*} \eta^{*} }}^{*} \frac{{\partial v^{*} }}{{\partial \xi^{*} }} \\ & \quad + \tau_{{\xi^{*} \eta^{*} }}^{*} \left( {\frac{{\partial u^{*} }}{{\partial \eta^{*} }} + \frac{{\partial v^{*} }}{{\partial \xi^{*} }}} \right) + \frac{{\sigma B_{0}^{2} }}{{\left[ {\left( {1 + \beta_{e} \beta_{i} } \right)^{2} + \beta_{e}^{2} } \right]}}\left( {\left( {u^{*} } \right)^{2} + \left( {v^{*} } \right)^{2} } \right) \\ & \quad + \left( {\rho C_{p} } \right)_{p} \left[ {\frac{{D_{T} }}{{T_{m} }}\left( {\left( {\frac{\partial T}{{\partial \xi^{*} }}} \right)^{2} + \left( {\frac{\partial T}{{\partial \eta^{*} }}} \right)^{2} } \right) + D_{B} \left( {\frac{\partial T}{{\partial \xi^{*} }}\frac{\partial C}{{\partial \xi^{*} }} + \frac{\partial T}{{\partial \eta^{*} }}\frac{\partial C}{{\partial \eta^{*} }}} \right)} \right] \\ \end{aligned}$$

7 $$\frac{\partial C}{{\partial t^{*} }} + u^{*} \frac{\partial C}{{\partial \xi^{*} }} + v^{*} \frac{\partial C}{{\partial \eta^{*} }} = D_{B} \left( {\frac{{\partial^{2} C}}{{\partial \xi^{*2} }} + \frac{{\partial^{2} C}}{{\partial \eta^{*2} }}} \right) + \frac{{D_{T} }}{{T_{m} }}\left[ {\left( {\frac{{\partial^{2} T}}{{\partial \xi^{*2} }}} \right) + \left( {\frac{{\partial^{2} T}}{{\partial \eta^{*2} }}} \right)} \right] - K_{1} \left( {C - C_{0} } \right)$$

Introducing the following transformations for dimensionless

8 $$\begin{aligned} u & = \frac{{u^{*} }}{s},\quad v = \frac{{v^{*} }}{s},\quad t = \frac{{s\,t^{*} }}{\lambda },\quad \xi = \frac{{\xi^{*} }}{\lambda },\quad \eta = \frac{{\eta^{*} }}{\lambda },\quad \tau = \frac{{d\tau^{*} }}{{s\mu_{f} }}\delta = \frac{d}{\lambda },\quad h_{1} = \frac{{H_{1} }}{d}, \\ h_{2} & = \frac{{H_{2} }}{d},\quad a = \frac{{a_{1} }}{d},\quad b = \frac{{a_{2} }}{d},\quad p = \frac{{p^{*} d^{2} }}{{\mu_{f} \lambda s}},\quad m = \frac{{\lambda m^{*} }}{d},\quad \theta = \frac{{T - T_{0} }}{{T_{1} - T_{0} }},\quad \phi = \frac{{C - C_{0} }}{{C_{1} - C_{0} }} \\ \end{aligned}$$

In view of relations (6), Eqs. (4)–(7) take the following form

9 $$\begin{aligned} & R_{a} \left( {\delta \frac{\partial u}{{\partial t}} + \delta u\frac{\partial u}{{\partial \xi }} + \delta v\frac{\partial u}{{\partial \eta }}} \right) = - \frac{\partial p}{{\partial \xi }} + \delta \frac{{\partial \tau_{\xi \xi } }}{\partial \xi } + \frac{{\partial \tau_{\xi \eta } }}{\partial \eta } + \frac{{M^{2} \left( {\beta_{e} v - \left( {1 + \beta_{e} \beta_{i} } \right)u} \right)}}{{\left( {1 + \beta_{e} \beta_{i} } \right)^{2} + \beta_{e}^{2} }} \\ & \quad + G_{r} \theta + G_{c} \phi + \frac{{R_{a} }}{{F_{r} }}\sin \Lambda , \\ \end{aligned}$$

10 $$\begin{aligned} & R_{a} \left( {\delta^{3} \frac{\partial v}{{\partial t}} + \delta^{3} u\frac{\partial v}{{\partial \xi }} + \delta^{3} v\frac{\partial v}{{\partial \eta }}} \right) = - \frac{\partial p}{{\partial \eta }} + \delta^{2} \frac{{\partial \tau_{\xi \eta } }}{\partial \xi } - \frac{{M^{2} \delta \left( {\beta_{e} u - \left( {1 + \beta_{e} \beta_{i} } \right)v} \right)}}{{\left( {1 + \beta_{e} \beta_{i} } \right)^{2} + \beta_{e}^{2} }} \\ & \quad + \delta \frac{{\partial \tau_{\eta \eta } }}{\partial \eta } - \frac{{\delta R_{a} }}{{F_{r} }}\cos \Lambda , \\ \end{aligned}$$

11 $$\begin{aligned} & R_{a} \left( {\delta \frac{\partial \theta }{{\partial t}} + \delta u\frac{\partial \theta }{{\partial \xi }} + \delta v\frac{\partial \theta }{{\partial \eta }}} \right) = \frac{{\delta^{2} }}{\Pr }\frac{{\partial^{2} \theta }}{{\partial \xi^{2} }} + \frac{1}{\Pr }\frac{{\partial^{2} \theta }}{{\partial \eta^{2} }} + N_{T} \delta^{2} \left( {\frac{\partial \theta }{{\partial \xi }}} \right)^{2} + N_{T} \left( {\frac{\partial \theta }{{\partial \eta }}} \right)^{2} + N_{B} \delta^{2} \frac{\partial \phi }{{\partial \xi }}\frac{\partial \theta }{{\partial \xi }} \\ & \quad + N_{B} \frac{\partial \phi }{{\partial \eta }}\frac{\partial \theta }{{\partial \eta }} + E_{C} \frac{{M^{2} }}{{\left( {1 + \beta_{e} \beta_{i} } \right)^{2} + \beta_{e}^{2} }}u^{2} + E_{C} \delta {\mkern 1mu} \tau_{\xi \xi } \frac{\partial u}{{\partial \xi }} + E_{C} \tau_{\xi \eta } \left( {\frac{\partial u}{{\partial \eta }} + \delta^{2} \frac{\partial v}{{\partial \xi }}} \right) + E_{C} \delta \tau_{\eta \eta } \frac{\partial v}{{\partial \eta }}, \\ \end{aligned}$$

12 $$R_{a} Sc\left( {\delta \frac{\partial \phi }{{\partial t}} + \delta u\frac{\partial \phi }{{\partial \xi }} + \delta v\frac{\partial \phi }{{\partial \eta }}} \right) = \delta^{2} \frac{{\partial^{2} \phi }}{{\partial \xi^{2} }} + \frac{{\partial^{2} \phi }}{{\partial \eta^{2} }} + \frac{{N_{T} }}{{N_{B} }}\left( {\delta^{2} \frac{{\partial^{2} \theta }}{{\partial \xi^{2} }} + \frac{{\partial^{2} \theta }}{{\partial \eta^{2} }}} \right) - K_{R} Sc{\mkern 1mu} \phi$$

where, $N_{B} = \frac{{\left( {\rho C_{P} } \right)_{p} }}{{\left( {\rho C_{P} } \right)_{f} }}\frac{{D_{B} \left( {C_{1} - C_{0} } \right)}}{{\nu_{f} }}$ is the Brownian motion, $N_{T} = \frac{{\left( {\rho C_{P} } \right)_{p} }}{{\left( {\rho C_{P} } \right)_{f} }}\frac{{D_{T} \left( {T_{1} - T_{0} } \right)}}{{\nu_{f} T_{m} }}$ ,$\Pr = \frac{{\mu_{f} \left( {C_{P} } \right)_{f} }}{{k_{f} }}$ is the Prandtl number, $E_{C} = \frac{{s^{2} }}{{\left( {C_{p} } \right)_{f} \left( {T_{1} - T_{0} } \right)}}$ is the Eckert number,$\,Sc = \frac{{\nu_{f} }}{{D_{B} }}$ is the Schmidt number,$K_{R} = \frac{{K_{r} d^{2} }}{{\nu_{f} }}$ is the chemical reaction parameter ,$K = \frac{{K_{a} }}{{d^{2} }}$ is the permeability parameter,$\,{\text{R}}_{{_{a} }} = \frac{{sd\rho_{f} }}{{\mu_{f} }}$ is the Reynolds number,$F_{r} = \frac{{s^{2} }}{gd}$ Froude number, $\gamma_{1} = \frac{{\sigma_{e} d^{2} E_{{\xi^{*} }}^{2} }}{{k_{f} \nabla T}}$ joule heating parameter.

By employing stream functions $u = \frac{\partial \psi }{{\partial \eta }}\,{\text{and}}\,v = - \frac{\partial \psi }{{\partial \xi }}$ smaller Reynolds number and larger-wavelength theory, the Eqs. (9)–(12) take the form

13 $$\frac{\partial p}{{\partial \xi }} = \frac{{\partial^{3} \psi }}{{\partial \eta^{3} }}\left( {1 - \beta_{F} \left( {\frac{{\partial^{2} \psi }}{{\partial \eta^{2} }}} \right)^{2} } \right) - \frac{{M^{2} \left( {1 + \beta_{e} \beta_{i} } \right)}}{{\left[ {\left( {1 + \beta_{e} \beta_{i} } \right)^{2} + \beta_{e}^{2} } \right]}}\frac{\partial \psi }{{\partial \eta }} + G_{R} \theta + G_{C} \phi + \frac{{R_{a} }}{{F_{r} }}\sin \Lambda = 0$$

Elimination of pressure

14 $$\frac{{\partial^{4} \psi }}{{\partial \eta^{4} }}\left( {1 - \beta_{F} \left( {\frac{{\partial^{2} \psi }}{{\partial \eta^{2} }}} \right)^{2} } \right) - \frac{{M^{2} \left( {1 + \beta_{e} \beta_{i} } \right)}}{{\left[ {\left( {1 + \beta_{e} \beta_{i} } \right)^{2} + \beta_{e}^{2} } \right]}}\frac{{\partial^{2} \psi }}{{\partial \eta^{2} }} + G_{R} \frac{\partial \theta }{{\partial \eta }} + G_{C} \frac{\partial \phi }{{\partial \eta }} = 0$$

15 $$\begin{aligned} & \frac{1}{\Pr }\frac{{\partial^{2} \theta }}{{\partial \eta^{2} }} + N_{T} \left( {\frac{\partial \theta }{{\partial \eta }}} \right)^{2} + N_{B} \frac{\partial \theta }{{\partial \eta }}\frac{\partial \phi }{{\partial \eta }} + E_{C} \frac{{M^{2} }}{{\left[ {\left( {1 + \beta_{e} \beta_{i} } \right)^{2} + \beta_{e}^{2} } \right]}}\left( {\frac{\partial \psi }{{\partial \eta }}} \right)^{2} \\ & \quad + E_{C} \left( {\frac{{\partial^{2} \psi }}{{\partial \eta^{2} }}} \right)^{2} \left( {1 - \beta_{F} \left( {\frac{{\partial^{2} \psi }}{{\partial \eta^{2} }}} \right)^{2} } \right) = 0 \\ \end{aligned}$$

16 $$\frac{{\partial^{2} \phi }}{{\partial \eta^{2} }} + \frac{{N_{T} }}{{N_{B} }}\frac{{\partial^{2} \theta }}{{\partial \eta^{2} }} - K_{R} Sc\,\phi = 0$$

$$\tau_{\xi \eta } = \left[ {1 - \beta_{F} \left( {\frac{{\partial^{2} \psi }}{{\partial \eta^{2} }}} \right)^{2} } \right]\frac{{\partial^{2} \psi }}{{\partial \eta^{2} }}$$

Vertical asymmetric walls have the following boundary conditions

17 $$\begin{aligned} \eta & = h_{2} = 1 + m\xi + b\sin \left( {2\pi \left( {\xi - t} \right)} \right),\,\psi = \frac{F}{2},\,\frac{\partial \psi }{{\partial \eta }} = 0,\theta = 1,\,\phi = 1 \\ \eta & = h_{1} = - 1 - m\xi - a\sin \left( {2\pi \left( {\xi - t} \right) + \omega } \right),\,\psi = - \frac{F}{2},\,\frac{\partial \psi }{{\partial \eta }} = 0,\,\,\theta = 0,\,\phi = 0 \\ \end{aligned}$$

Non-dimension flow rate is given as

$$Q = F + d + 1$$

In which, $$F\left( {\xi ,t} \right) = \Theta + a\sin \left( {2\pi \left( {\xi - t} \right) + \omega } \right) + b\sin \left( {2\pi \left( {\xi - t} \right)} \right)$$.

Where, a & b amplitudes of left and right walls, m is the non-uniform parameter, $\omega$ is the phase difference.

## Homotopy perturbation solution

### Analysis of the HPM

The steps that might be taken to create a homotopy perturbation method are outlined below.

Considering the following equations

18 $$\Im (\varpi ) - \ell (z) = 0,\;\;z \in \psi$$

Considering the limiting conditions

19 $$\dag (\varpi ,\frac{\partial \varpi }{{\partial n}}) = 0,\;\;z \in \partial \psi$$

The variable $\Im$ is partitioned into two binary components, a linear component denoted as $L$ and a nonlinear component denoted as $N$.

20 $$L\left( \varpi \right) + N\left( \varpi \right) = \ell (z)$$

The following is an explanation of how the construction of the HPM is presented

21 $$G\left( {\varpi ,\eta } \right) = \left( {1 - P} \right)\;\left[ {L\left( \varpi \right) - L\left( {\varpi_{0} } \right)} \right] + P\left[ {\Im (\varpi ) - \ell (z)} \right] = 0$$

where,

22 $$\varpi \left( {z,\eta } \right):\psi \times \left[ {0,1} \right] \to S_{1}$$

### The execution of the HPM technique

The suitable boundary conditions and associated nonlinear dimensionless equations are calculated using HPM.

The following sequence of functions are presented in order to use this method

23 $$\psi \left( \eta \right) = \psi_{0} \left( \eta \right) + p\psi_{1} \left( \eta \right) + p^{2} \psi_{2} \left( \eta \right) + \cdots$$

24 $$\theta \left( \eta \right) = \theta_{0} \left( \eta \right) + p\theta_{1} \left( \eta \right) + p^{2} \theta_{2} \left( \eta \right) + \cdots$$

25 $$\phi \left( \eta \right) = \phi_{0} \left( \eta \right) + p\phi_{1} \left( \eta \right) + p^{2} \phi_{2} \left( \eta \right) + \cdots$$

By substituting the above expression in the proposed model we get the following system

26 $$\psi : = {\mkern 1mu} \left( {\begin{array}{*{20}l} {{\mkern 1mu} {\mkern 1mu} \left( {1 - p} \right)\left( {\frac{{d^{4} }}{{d\eta^{4} }}\psi_{0} \left( \eta \right) + p\frac{{d^{4} }}{{d\eta^{4} }}\psi_{1} \left( \eta \right) + p^{2} \frac{{d^{4} }}{{d\eta^{4} }}\psi_{2} \left( \eta \right)} \right)} \hfill \\ {{\mkern 1mu} {\mkern 1mu} + p\left( {\begin{array}{*{20}l} {\left( {\frac{{d^{4} }}{{d\eta^{4} }}\psi_{0} \left( \eta \right) + p\frac{{d^{4} }}{{d\eta^{4} }}\psi_{1} \left( \eta \right) + p^{2} \frac{{d^{4} }}{{d\eta^{4} }}\psi_{2} \left( \eta \right)} \right)} \hfill \\ {\left( {1 - \beta_{F} \left( {\frac{{d^{2} }}{{d\eta^{2} }}\psi_{0} \left( \eta \right) + p\frac{{d^{2} }}{{d\eta^{2} }}\psi_{1} \left( \eta \right) + p^{2} \frac{{d^{2} }}{{d\eta^{2} }}\psi_{2} \left( \eta \right)} \right)^{2} } \right)} \hfill \\ { - \frac{{M^{2} \left( {\beta_{e} \beta_{i} + 1} \right)\left( {\frac{{d^{2} }}{{d\eta^{2} }}\psi_{0} \left( \eta \right) + p\frac{{d^{2} }}{{d\eta^{2} }}\psi_{1} \left( \eta \right) + p^{2} \frac{{d^{2} }}{{d\eta^{2} }}\psi_{2} \left( \eta \right)} \right)}}{{\left( {\beta_{e} \beta_{i} + 1} \right)^{2} + \beta_{e}^{2} }}} \hfill \\ { + G_{R} \left( {\frac{d}{d\eta }\theta_{0} \left( \eta \right) + p\frac{d}{d\eta }\theta_{1} \left( \eta \right) + p^{2} \frac{d}{d\eta }\theta_{2} \left( \eta \right)} \right)} \hfill \\ { + G_{C} \left( {\frac{d}{d\eta }f_{0} \left( \eta \right) + p\frac{d}{d\eta }f_{1} \left( \eta \right) + p^{2} \frac{d}{d\eta }f_{2} \left( \eta \right)} \right)} \hfill \\ \end{array} } \right)} \hfill \\ \end{array} } \right){\mkern 1mu} {\mkern 1mu} {\mkern 1mu} {\mkern 1mu} {\mkern 1mu} {\mkern 1mu} {\mkern 1mu} {\mkern 1mu} {\mkern 1mu} {\mkern 1mu} {\mkern 1mu}$$

27 $$\theta : = \left( \begin{gathered} \left( {1 - p} \right)\left( {\frac{{{\text{d}}^{2} }}{{{\text{d}}\eta^{2} }}\theta_{0} \left( \eta \right) + p\frac{{{\text{d}}^{2} }}{{{\text{d}}\eta^{2} }}\theta_{1} \left( \eta \right) + p^{2} \frac{{{\text{d}}^{2} }}{{{\text{d}}\eta^{2} }}\theta_{2} \left( \eta \right)} \right) \hfill \\ + p\left( \begin{gathered} + N_{B} \left( {\frac{{\text{d}}}{{{\text{d}}\eta }}\theta_{0} \left( \eta \right) + p\frac{{\text{d}}}{{{\text{d}}\eta }}\theta_{1} \left( \eta \right) + p^{2} \frac{{\text{d}}}{{{\text{d}}\eta }}\theta_{2} \left( \eta \right)} \right) \hfill \\ \,\,\,\,\,\,\,\,\,\,\,\,\,\,\,\,\,\,\,\,\left( {\frac{{\text{d}}}{{{\text{d}}\eta }}\phi_{0} \left( \eta \right) + p\frac{{\text{d}}}{{{\text{d}}\eta }}\phi_{1} \left( \eta \right) + p^{2} \frac{{\text{d}}}{{{\text{d}}\eta }}\phi_{2} \left( \eta \right)} \right) \hfill \\ + \frac{{\frac{{{\text{d}}^{2} }}{{{\text{d}}\eta^{2} }}\theta_{0} \left( \eta \right) + p\frac{{{\text{d}}^{2} }}{{{\text{d}}\eta^{2} }}\theta_{1} \left( \eta \right) + p^{2} \frac{{{\text{d}}^{2} }}{{{\text{d}}\eta^{2} }}\theta_{2} \left( \eta \right)}}{\Pr } \hfill \\ + E_{C} \left( {\frac{{{\text{d}}^{2} }}{{{\text{d}}\eta^{2} }}\psi_{0} \left( \eta \right) + p\frac{{{\text{d}}^{2} }}{{{\text{d}}\eta^{2} }}\psi_{1} \left( \eta \right) + p^{2} \frac{{{\text{d}}^{2} }}{{{\text{d}}\eta^{2} }}\psi_{2} \left( \eta \right)} \right)^{2} \hfill \\ \left( {1 - \beta_{F} \left( {\frac{{{\text{d}}^{2} }}{{{\text{d}}\eta^{2} }}\psi_{0} \left( \eta \right) + p\frac{{{\text{d}}^{2} }}{{{\text{d}}\eta^{2} }}\psi_{1} \left( \eta \right) + p^{2} \frac{{{\text{d}}^{2} }}{{{\text{d}}\eta^{2} }}\psi_{2} \left( \eta \right)} \right)^{2} } \right) \hfill \\ + N_{T} \left( {\frac{{\text{d}}}{{{\text{d}}\eta }}\theta_{0} \left( \eta \right) + p\frac{{\text{d}}}{{{\text{d}}\eta }}\theta_{1} \left( \eta \right) + p^{2} \frac{{\text{d}}}{{{\text{d}}\eta }}\theta_{2} \left( \eta \right)} \right)^{2} \hfill \\ + \frac{{E_{C} M^{2} \left( {\frac{{\text{d}}}{{{\text{d}}\eta }}\psi_{0} \left( \eta \right) + p\frac{{\text{d}}}{{{\text{d}}\eta }}\psi_{1} \left( \eta \right) + p^{2} \frac{{\text{d}}}{{{\text{d}}\eta }}\psi_{2} \left( \eta \right)} \right)^{2} }}{{\left( {\beta_{e} \beta_{i} + 1} \right)^{2} + \beta_{e}^{2} }} \hfill \\ \end{gathered} \right) \hfill \\ \end{gathered} \right)$$

28 $$\phi : = \left( {\begin{array}{*{20}l} {\left( {1 - p} \right)\left( {\frac{{{\text{d}}^{2} }}{{{\text{d}}\eta^{2} }}\phi_{0} \left( \eta \right) + p\frac{{{\text{d}}^{2} }}{{{\text{d}}\eta^{2} }}\phi_{1} \left( \eta \right) + p^{2} \frac{{{\text{d}}^{2} }}{{{\text{d}}\eta^{2} }}\phi_{2} \left( \eta \right)} \right)} \hfill \\ { + p\left( {\begin{array}{*{20}l} { + \frac{{N_{T} }}{{N_{B} }}\left( {\frac{{{\text{d}}^{2} }}{{{\text{d}}\eta^{2} }}\theta_{0} \left( \eta \right) + p\frac{{{\text{d}}^{2} }}{{{\text{d}}\eta^{2} }}\theta_{1} \left( \eta \right) + p^{2} \frac{{{\text{d}}^{2} }}{{{\text{d}}\eta^{2} }}\theta_{2} \left( \eta \right)} \right)} \hfill \\ {\frac{{{\text{d}}^{2} }}{{{\text{d}}\eta^{2} }}\phi_{0} \left( \eta \right) + p\frac{{{\text{d}}^{2} }}{{{\text{d}}\eta^{2} }}\phi_{1} \left( \eta \right) + p^{2} \frac{{{\text{d}}^{2} }}{{{\text{d}}\eta^{2} }}\phi_{2} \left( \eta \right)} \hfill \\ { - ScK_{R} \left( {\phi_{0} \left( \eta \right) + p\phi_{1} \left( \eta \right) + p^{2} \phi_{2} \left( \eta \right)} \right)} \hfill \\ \end{array} } \right)} \hfill \\ \end{array} } \right)$$

Zeroth-order procedure

29 $$\frac{{{\text{d}}^{4} }}{{{\text{d}}\eta^{4} }}\psi_{0} \left( \eta \right) = 0;\quad \frac{{{\text{d}}^{2} }}{{{\text{d}}\eta^{2} }}\theta_{0} \left( \eta \right) = 0;\quad \frac{{{\text{d}}^{2} }}{{{\text{d}}\eta^{2} }}\phi_{0} \left( \eta \right) = 0$$

First-order procedure

30 $$\begin{aligned} & \frac{{d^{4} }}{{d\eta^{4} }}\psi_{1} \left( \eta \right) - \frac{{d^{4} }}{{d\eta^{4} }}\psi_{0} \left( \eta \right) + \left( {\frac{{d^{4} }}{{d\eta^{4} }}\psi_{0} \left( \eta \right)} \right)\left( {1 - \beta_{F} \left( {\frac{{d^{2} }}{{d\eta^{2} }}\psi_{0} \left( \eta \right)} \right)^{2} } \right) + G_{R} \frac{d}{d\eta }\theta_{0} \left( \eta \right) \\ & \quad - \frac{{M^{2} \left( {\beta_{1} \beta_{e} + 1} \right)\frac{{d^{2} }}{{d\eta^{2} }}\psi_{0} \left( \eta \right)}}{{\left( {\beta_{1} \beta_{e} + 1} \right)^{2} + \beta_{e}^{2} }} + G_{C} \frac{d}{d\eta }\phi_{0} \left( \eta \right) = 0 \\ \end{aligned}$$

31 $$\begin{aligned} & \frac{{{\text{d}}^{2} }}{{{\text{d}}\eta^{2} }}\theta_{1} \left( \eta \right) - \frac{{{\text{d}}^{2} }}{{{\text{d}}\eta^{2} }}\theta_{0} \left( \eta \right) + \frac{{\frac{{{\text{d}}^{2} }}{{{\text{d}}\eta^{2} }}\theta_{0} \left( \eta \right)}}{\Pr } + N_{T} \left( {\frac{{\text{d}}}{{{\text{d}}\eta }}\theta_{0} \left( \eta \right)} \right)^{2} + N_{B} \left( {\frac{{\text{d}}}{{{\text{d}}\eta }}\theta_{0} \left( \eta \right)} \right)\frac{{\text{d}}}{{{\text{d}}\eta }}\phi_{0} \left( \eta \right) \\ & \quad + E_{C} \left( {\frac{{{\text{d}}^{2} }}{{{\text{d}}\eta^{2} }}\psi_{0} \left( \eta \right)} \right)^{2} \left( {1 - \beta_{F} \left( {\frac{{{\text{d}}^{2} }}{{{\text{d}}\eta^{2} }}\psi_{0} \left( \eta \right)} \right)^{2} } \right) + \frac{{E_{C} M^{2} \left( {\frac{{\text{d}}}{{{\text{d}}\eta }}\psi_{0} \left( \eta \right)} \right)^{2} }}{{\left( {\beta_{1} \beta_{e} + 1} \right)^{2} + \beta_{e}^{2} }} = 0 \\ \end{aligned}$$

32 $$\frac{{{\text{d}}^{2} }}{{{\text{d}}\eta^{2} }}\phi_{1} \left( \eta \right) + \frac{{N_{T} \frac{{{\text{d}}^{2} }}{{{\text{d}}\eta^{2} }}\theta_{0} \left( \eta \right)}}{{N_{B} }} - ScK_{R} \phi_{0} \left( \eta \right) = 0$$

Second-order procedure

33 $$\begin{aligned} & \frac{{{\text{d}}^{4} }}{{{\text{d}}\eta^{4} }}\psi_{2} \left( \eta \right) - \frac{{{\text{d}}^{4} }}{{{\text{d}}\eta^{4} }}\psi_{1} \left( \eta \right) - 2\left( {\frac{{{\text{d}}^{4} }}{{{\text{d}}\eta^{4} }}\psi_{0} \left( \eta \right)} \right)\beta_{F} \left( {\frac{{{\text{d}}^{2} }}{{{\text{d}}\eta^{2} }}\psi_{0} \left( \eta \right)} \right)\frac{{{\text{d}}^{2} }}{{{\text{d}}\eta^{2} }}\psi_{1} \left( \eta \right) + G_{R} \frac{{\text{d}}}{{{\text{d}}\eta }}\theta_{1} \left( \eta \right) \\ & \quad + \left( {\frac{{{\text{d}}^{4} }}{{{\text{d}}\eta^{4} }}\psi_{1} \left( \eta \right)} \right)\left( {1 - \beta_{F} \left( {\frac{{{\text{d}}^{2} }}{{{\text{d}}\eta^{2} }}\psi_{0} \left( \eta \right)} \right)^{2} } \right) - \frac{{M^{2} \left( {\beta_{2} \beta_{e} + 1} \right)\frac{{{\text{d}}^{2} }}{{{\text{d}}\eta^{2} }}\psi_{1} \left( \eta \right)}}{{\left( {\beta_{2} \beta_{e} + 1} \right)^{2} + \beta_{e}^{2} }} + G_{C} \frac{{\text{d}}}{{{\text{d}}\eta }}\phi_{1} \left( \eta \right) = 0 \\ \end{aligned}$$

34 $$\begin{gathered} \frac{{{\text{d}}^{2} }}{{{\text{d}}\eta^{2} }}\theta_{2} \left( \eta \right) - \frac{{{\text{d}}^{2} }}{{{\text{d}}\eta^{2} }}\theta_{1} \left( \eta \right) + \frac{{\frac{{{\text{d}}^{2} }}{{{\text{d}}\eta^{2} }}\theta_{1} \left( \eta \right)}}{\Pr } + 2N_{T} \left( {\frac{{\text{d}}}{{{\text{d}}\eta }}\theta_{0} \left( \eta \right)} \right)\frac{{\text{d}}}{{{\text{d}}\eta }}\theta_{1} \left( \eta \right) \hfill \\ + N_{B} \left( {\frac{{\text{d}}}{{{\text{d}}\eta }}\theta_{0} \left( \eta \right)} \right)\frac{{\text{d}}}{{{\text{d}}\eta }}\phi_{1} \left( \eta \right) + N_{B} \left( {\frac{{\text{d}}}{{{\text{d}}\eta }}\theta_{1} \left( \eta \right)} \right)\frac{{\text{d}}}{{{\text{d}}\eta }}\phi_{0} \left( \eta \right) \hfill \\ - 2E_{C} \left( {\frac{{{\text{d}}^{2} }}{{{\text{d}}\eta^{2} }}\psi_{0} \left( \eta \right)} \right)^{3} \beta_{F} \frac{{{\text{d}}^{2} }}{{{\text{d}}\eta^{2} }}\psi_{1} \left( \eta \right) + 2\frac{{E_{C} M^{2} \left( {\frac{{\text{d}}}{{{\text{d}}\eta }}\psi_{0} \left( \eta \right)} \right)\frac{{\text{d}}}{{{\text{d}}\eta }}\psi_{1} \left( \eta \right)}}{{\left( {\beta_{2} \beta_{e} + 1} \right)^{2} + \beta_{e}^{2} }} \hfill \\ + 2E_{C} \left( {\frac{{{\text{d}}^{2} }}{{{\text{d}}\eta^{2} }}\psi_{0} \left( \eta \right)} \right)\left( {\frac{{{\text{d}}^{2} }}{{{\text{d}}\eta^{2} }}\psi_{1} \left( \eta \right)} \right)\left( {1 - \beta_{F} \left( {\frac{{{\text{d}}^{2} }}{{{\text{d}}\eta^{2} }}\psi_{0} \left( \eta \right)} \right)^{2} } \right) = 0 \hfill \\ \end{gathered}$$

35 $$\frac{{{\text{d}}^{2} }}{{{\text{d}}\eta^{2} }}\phi_{2} \left( \eta \right) + \frac{{N_{T} \frac{{{\text{d}}^{2} }}{{{\text{d}}\eta^{2} }}\theta_{1} \left( \eta \right)}}{{N_{B} }} - ScK_{R} \phi_{1} \left( \eta \right) = 0$$

By fixing $$\Pr \, : = \, 21; \, N_{B} \, : = \, 0.5; \, G_{C} \, : = \, 0.2; \, \beta_{F} \, : = \, 1; \, G_{R} \, : = \, 0.2; \, \beta_{i} \, : = \, 0.4; \, \beta_{e} \, : = 0 \, .5; \,$$ $$N_{T} \, : = \, 1; \, K_{R} \, : = \, .5; \, Sc \, : = 0 \, .6;\,\,\xi : = 0.4;\,t: = 0.2; \, E_{C} \, : = \, 0.1;\Theta : = 1.4; \, M \, : = \, 1;$$ $$\,a: = 0.3;$$$$\,b: = 0.3;\omega : = {\pi \mathord{\left/ {\vphantom {\pi 3}} \right. \kern-0pt} 3};$$ are obtained as follows by solving the series of functions.

$$\begin{aligned} & \psi : - 0.0557051150073254246 - 0.000002507211831{\mkern 1mu} \eta^{9} + 0.000000700415712498828533\eta^{8} \\ & \quad - 0.00159526762663897188\eta^{7} - 0.000437344682700783270{\mkern 1mu} \eta^{6} - 0.00590205595371576415\eta^{5} \\ & \quad - 0.00372810735110439993{\mkern 1mu} \eta^{4} - 0.163720970462385013{\mkern 1mu} \eta^{3} + 0.0392845599708364080{\mkern 1mu} \eta^{2} \\ & \quad + 1.03368242276481737\eta \\ \end{aligned}$$

$$\begin{aligned} & \theta :0.935680222799999960 + 0.001738482617{\mkern 1mu} \eta^{8} - 0.0000699205341099992380{\mkern 1mu} \eta^{7} \\ & \quad + 0.00793300027099999930{\mkern 1mu} \eta^{6} - 0.00254884465199998892{\mkern 1mu} \eta^{5} - 0.00966402786900004418{\mkern 1mu} \eta^{4} \\ & \quad + 0.0409078656200000668{\mkern 1mu} \eta^{3} - 0.271312562400000179{\mkern 1mu} \eta^{2} + 0.322176649299999951{\mkern 1mu} \eta \\ \end{aligned}$$

$$\begin{aligned} & \phi : - 0.0557051150073254246 - 0.000002507211831{\mkern 1mu} \eta^{9} + 0.000000700415712498828533{\mkern 1mu} \eta^{8} \\ & \quad - 0.00159526762663897188\eta^{7} - 0.000437344682700783270{\mkern 1mu} \eta^{6} - 0.00590205595371576415{\mkern 1mu} \eta^{5} \\ & \quad - 0.00372810735110439993\eta^{4} - 0.163720970462385013{\mkern 1mu} \eta^{3} + 0.0392845599708364080{\mkern 1mu} \eta^{2} \\ & \quad + 1.03368242276481737{\mkern 1mu} \eta \\ \end{aligned}$$

**Figure 2:**
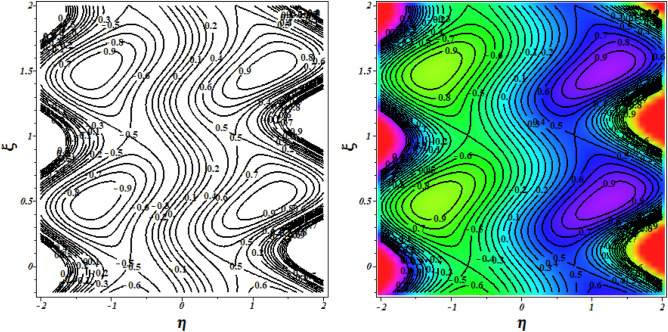
Code validation with Kothandapani and Prakash^39^ when m = 0.

**Figure 3:**
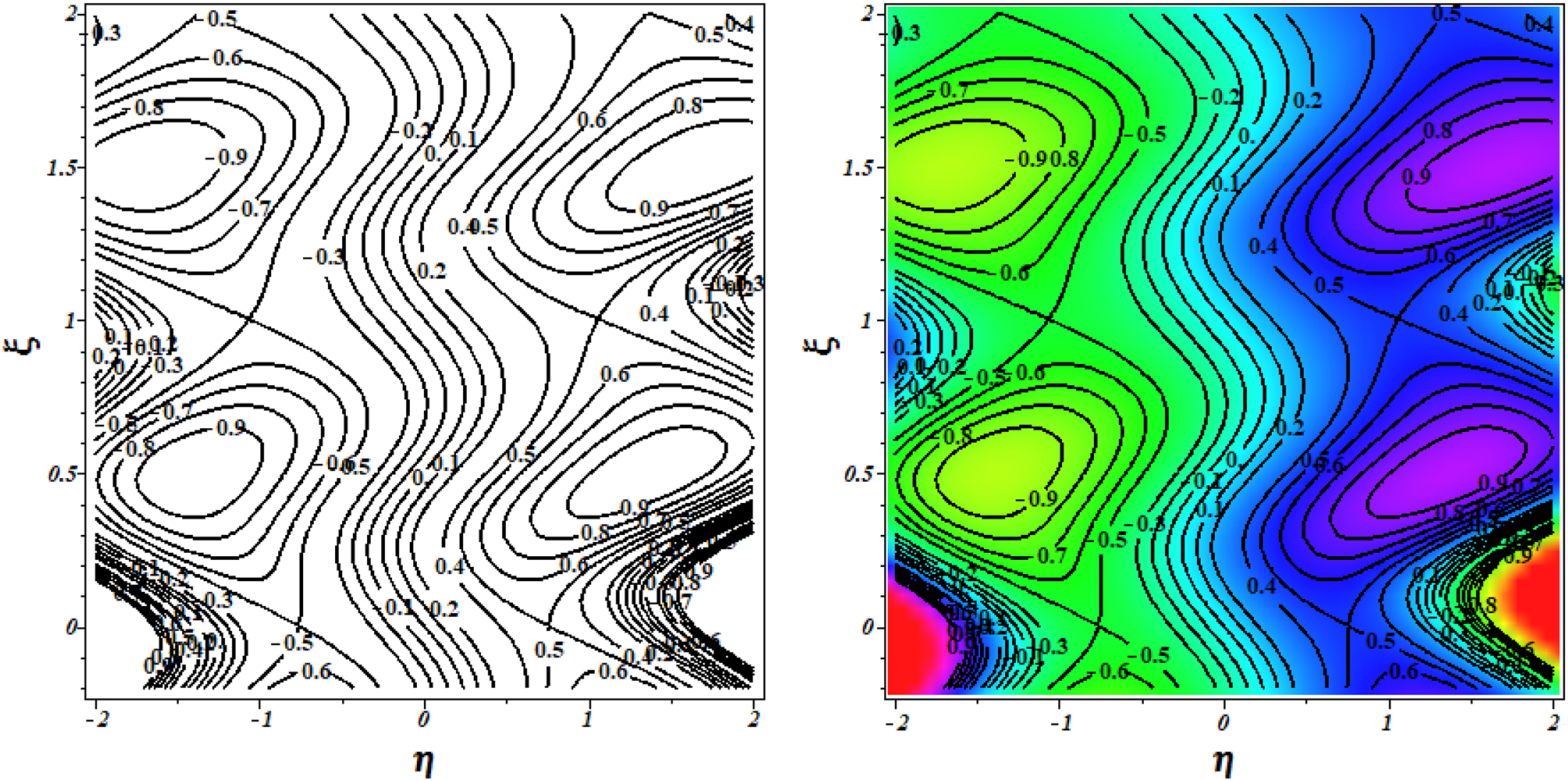
Code validation with Kothandapani and Prakash^39^ when m = 0.3.

To verify the precision of the code, we conduct a comparison between the streamlines of the current problem and the outcomes reported by Kothandapani and Prakash ^39^. For this comparison, we set the values of $\Theta = 1.5,\,M = 2,\,a = 0.3,\,\omega = {\pi \mathord{\left/ {\vphantom {\pi 2}} \right. \kern-0pt} 2},\,Sc = 0,\,\Pr = 0.7,\,G_{r} = 1.5,\,G_{c} = 1,\,t = 0.4,b = 0.4,\,N_{B} = 2,\,N_{T} = 1,\,$, and the results are presented in Figs. 2 and 3.

**Figure 4:**
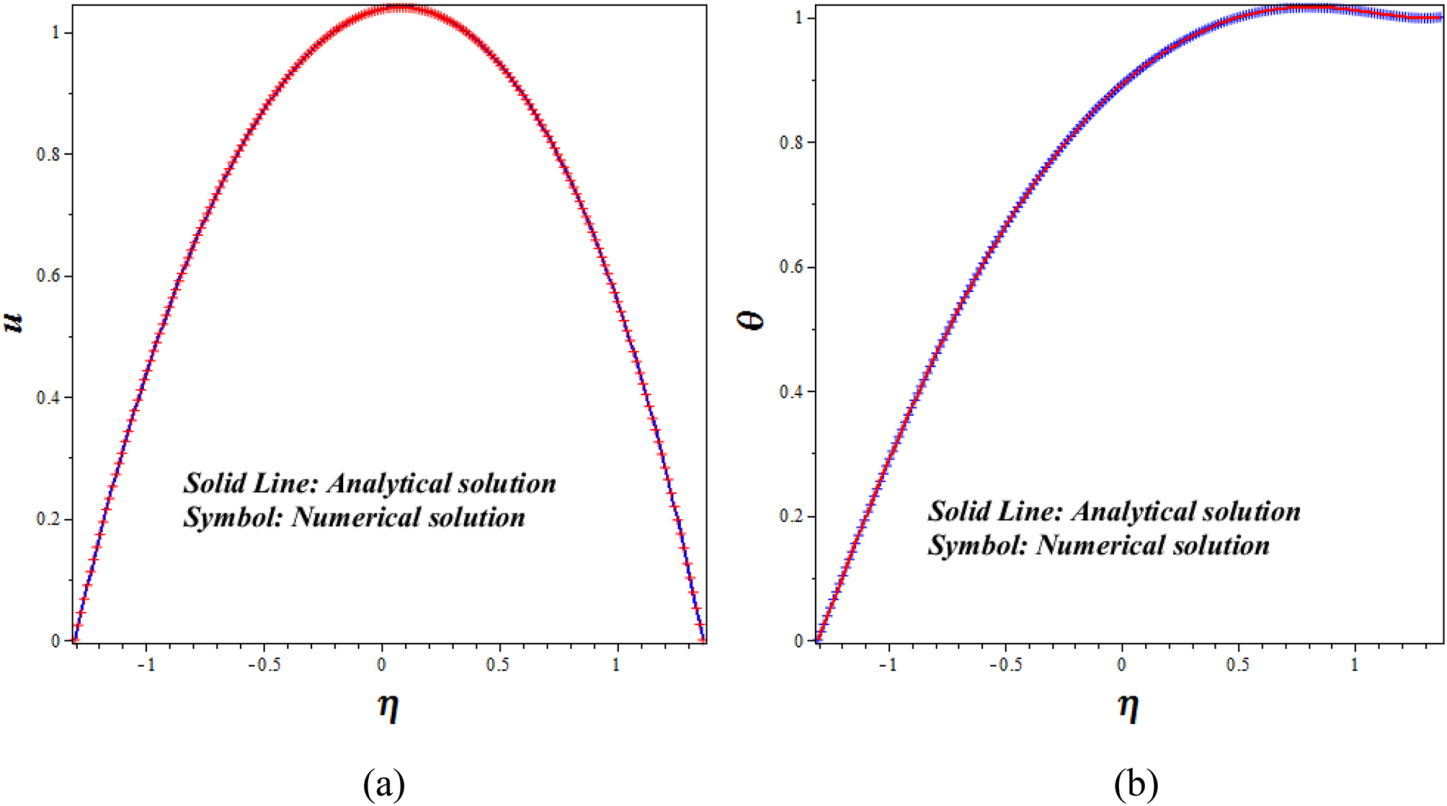
Code validation with numerical method on (**a**) Velocity (**b**) Temperature.

Upon analysis, it's evident that the current developments align with those of Kothandapani and Prakash^39^, showcasing similar patterns in fluid density trapping boluses. Smoothes with a value of 0.5 are where these boluses merge. Figure 4a, b compare the axial velocity and temperature from the HPM solution with the Runge–Kutta (RKF) approach, showing that the two methods are consistent with each other. This comparison demonstrates the feasibility and reliability of the current HPM solution.

## Artificial neural network modelling

Drawing inspiration from the intricate interconnections of neural cells in the human brain, the artificial neural network (ANN) stands as a cutting-edge computational paradigm that has gained prominence in recent years. When it comes to grouping, optimization, prediction, learning, classification, and generalization, it's on par with the human brain since it mimics the way neural networks in the brain have evolved over time^40^.

Significant merits of the ANN approach are articulated as follows:

• The ANN exhibits remarkable efficiency even when operating on minimal hardware configurations.

• It astonishingly simplifies the intricate mapping of complex classes.

• The desirable outcomes within the training set are governed by the input vector.

• Weights, symbolizing outcomes, are iteratively refined through training iterations.

**Figure 5:**
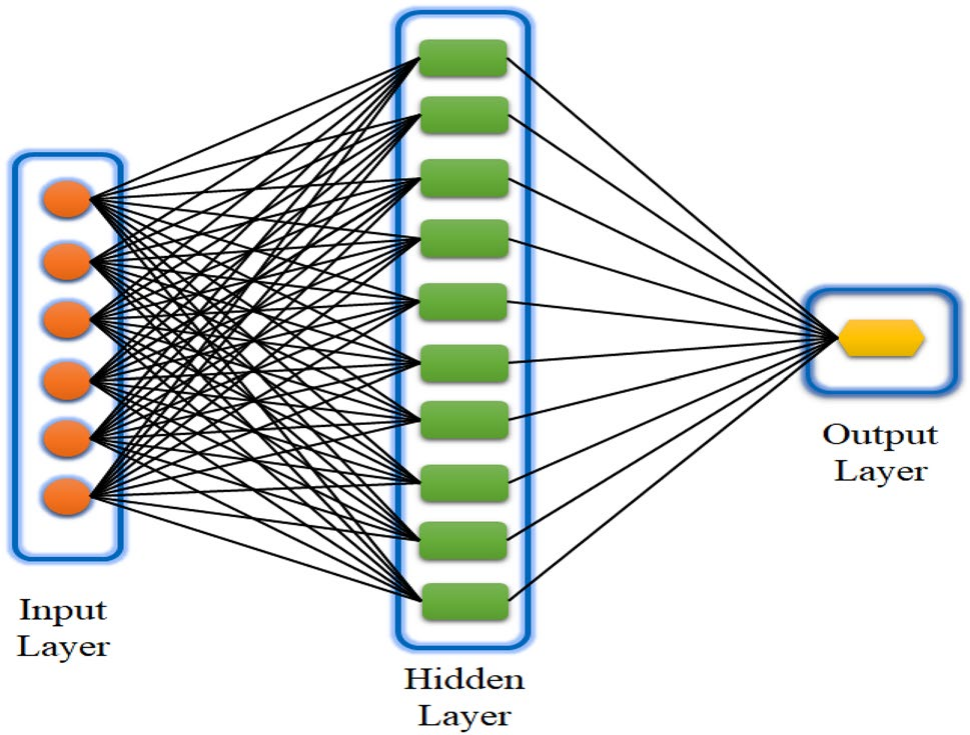
Schematic representation of multi-layer ANN model.

A wide variety of topologies are produced by combining training rules with neuron connections. In most cases, layers are formed as a result of the tight connections between neurons. There are essentially three levels to the design of an ANN: input, hidden, and output. These layers take data from the outside world, process it, and then send it on via the ANN. Without undergoing any processing at the input layer, data is sent directly to the neurons in the hidden layer. Translation is made easier by adjusting connecting lines, neuron interconnections, and weights. An ANN training database is kept by the system, which contains input values and their associated weights. Determining the ideal amount of layers and hidden neurons according to data use is one of the aspects that influence the creation of an ANN. One interesting and popular model in the field of artificial neural networks (ANN) right now is the feed-forward neural network (FFNN), which is based on the multi-layer perceptron architecture (MLP). The backpropagation approach is quite efficient compared to the other training techniques for FFNNs. This method deftly adjusts the weights of neurons as the network's output error is being computed, and then applies these changes consistently to all neurons in order to reduce the output error. The graphical representation of multi-layer ANN model is shown in Fig. 5

**Figure 6:**
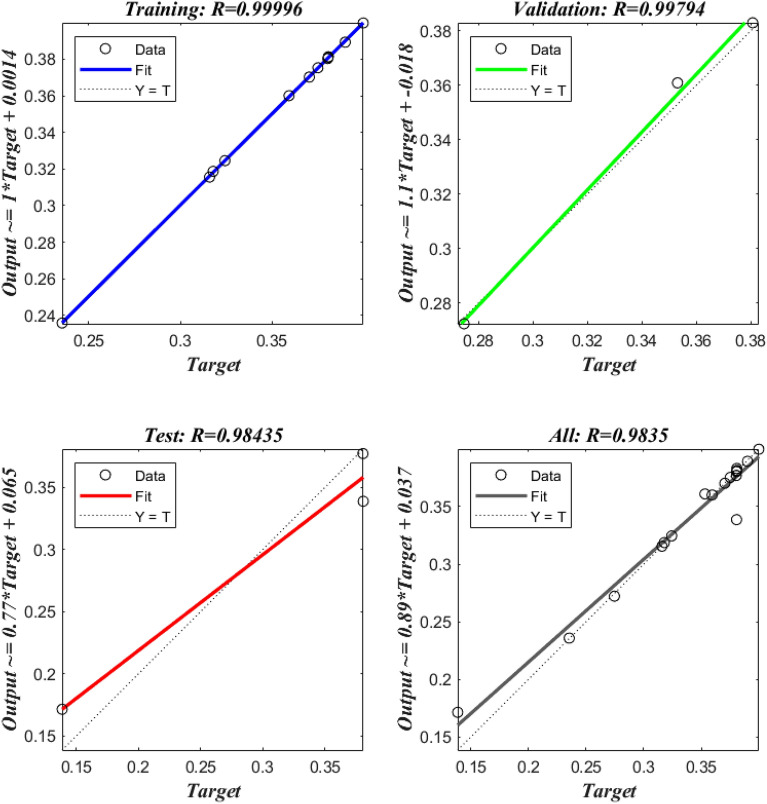
Graphical representation of the Nusselt number.

Finding the optimal hidden layer node count is an iterative process dependent on the total number of training epochs needed for the network. This makes sure the learning process doesn't go off course due to input parameter over- or under-configuration. After running the model through its paces, we found that using a single hidden layer consisting of five neurons significantly reduced the variation in the predicted Nu values. We trained the model on 70% of the dataset, validated it on 15%, and tested its predicting ability on the remaining 15%. Figure 6 displays the results of the ANN model's testing, validation, and training sets with respect to the skin friction coefficient and heat transfer rate. In order for ANN models to mimic complex relationships between input and output variables, this graphical representation is essential. The ANN model's outputs are quite congruent with computationally-derived values. Supplementary insights into heat transfer rate across various parameters are provided in Table 1, with the findings of the ANN model consistently complementing numerical outcomes. This investigation convincingly demonstrates the high-precision predictive capability of the ANN in estimating heat transfer rates.

**Table 1:** HPM solution and ANN values on$Nu$.

$N_{T}$	$N_{B}$	$M$	$\beta_{e}$	$\beta_{i}$	$\beta_{F}$	$Nu$	ANN	Error
1.5	0.5	1	0.1	0.1	1	0.315924	0.315589	0.000335
2	0.5	1	0.1	0.1	1	0.235448	0.235875	−0.00043
2.5	0.5	1	0.1	0.1	1	0.138782	0.171534	−0.03275
1	1	1	0.1	0.1	1	0.353109	0.360916	−0.00781
1	1.5	1	0.1	0.1	1	0.317912	0.31871	−0.0008
1	2	1	0.1	0.1	1	0.274622	0.272281	0.002341
1	0.5	1.5	0.1	0.1	1	0.375097	0.375265	−0.00017
1	1	2	0.1	0.1	1	0.35933	0.360059	−0.00073
1	0.5	2.5	0.1	0.1	1	0.324302	0.324551	−0.00025
1	0.5	1	1	0.1	1	0.38038	0.376992	0.003387
1	0.5	1	2	0.1	1	0.3805	0.380314	0.000187
1	0.5	1	3	0.1	1	0.380577	0.338641	0.041936
1	0.5	1	0.1	1	1	0.380799	0.381286	−0.00049
1	0.5	1	0.1	2	1	0.380775	0.383018	−0.00224
1	0.5	1	0.1	3	1	0.38071	0.380908	−0.0002
1	0.5	1	0.1	0.1	0	0.370398	0.370201	0.000196
1	0.5	1	0.1	0.1	2	0.390023	0.389251	0.000772
1	0.5	1	0.1	0.1	3	0.399835	0.399723	0.000112

Mean Square error = 0.000000209144.

Levenberg–Marquardt was used to create the Multilayer Perceptron (MLP) feed-forward back-propagation ANN used in the smart numerical computing solver. The MLP-ANN model has input, hidden, and output layers and is based on the feed-forward architecture. The network was trained using the Levenberg–Marquardt algorithm, a powerful optimization strategy. During training, the weights of connections between neurons were tweaked repeatedly to reduce the discrepancy between the two sets of data. Reynolds number, Hall parameter, ion-slip parameter, Brownian motion, thermophoresis, inclination of the channel, and other important characteristics impacting the system are sent into the ANN's input layer. The hidden layer analyzes this input via weighted connections, and the output layer delivers predictions for variables of interest, such as velocity, temperature, and concentration. By adjusting the learning rate while optimizing, the Levenberg–Marquardt approach improves the ANN's training efficiency. This method expedites the convergence of the model and yields reliable results in prediction. In the context of magnetic viscoelastic nanofluid flow, the trained ANN acts as an intelligent solution, offering a computationally efficient alternative to conventional numerical approaches. The use of this smart numerical computer solution enhances the study's predictive power and applicability to other domains.

## Graphical illustrations and discussion

The purpose of this section is to depict the behavior of the magneto bio-Sutterby blood nanofluid by simulating its peristaltic flow in a hall-current and ion-slip-enhanced tapered inclined channel of key parameters, such as Reynold’s number $\left( {R_{a} = 0.5,1.0,1.5,2.0} \right)$, Froude number $\left( {F_{r} = 0.5,1.0,1.5,2.0} \right)$, Hartmann number $\left( {M^{2} = 1.0,1.5,2.0,2.5} \right)$, Hall parameter $\left( {\beta_{e} = 0.1,1.0,2.0,3.0} \right)$, ion-slip parameter $\left( {\beta_{i} = 0.1,1.0,2.0,3.0} \right)$,Brownian motion $\left( {N_{B} = 0.5,1.0,1.5,2.0} \right)$, thermophoresis $\left( {N_{T} = 0.5,1.0,1.5,2.0} \right)$, Eckert number $\left( {E_{c} = 0.0,0.1,0.2,0.3} \right)$, inclination of channel $\left( {\Lambda = \frac{\pi }{2},\frac{\pi }{3},\frac{\pi }{4},\frac{\pi }{5}} \right)$ and local temperature Grashof number $\left( {G_{R} = 1.0,2.0,3.0,4.0} \right)$ on bio-Sutterby blood nanofluid velocity $\left( u \right)$, pressure rise $\left( {\Delta p} \right)$, temperature $\left( \theta \right)$, heat transfer $\left( Z \right)$, concentration $\left( \phi \right)$, streamline and isothermal lines. The selection of active parameter values such as $\Pr = 21,$ $N_{B} = 0.5,\,$ $N_{T} = 1,\,$ $E_{c} = 0.1,\,$ $G_{R} = 0.2,\,$ $\beta_{i} = 0.4,\,$ $\beta_{e} = 0.5,\,$ $S_{C} = 0.6,\,$ $M = 2,\,$ $m = 0.2,\,$ $a = 0.3,\,b = 0.3,\,$ $t = 0.2,\,$ $\Theta = 1.4,\,$ $\omega = {\pi \mathord{\left/ {\vphantom {\pi 2}} \right. \kern-0pt} 2},$ $F_{r} = 0.5,\,$ and $R_{a} = 0.5$ both in terms of varying and maintaining fixed values, follows the methodology outlined in the works of Basha and Sivaraj^36^ as well as Kothandapani and Prakash^39^.

### Pressure gradient

**Figure 7:**
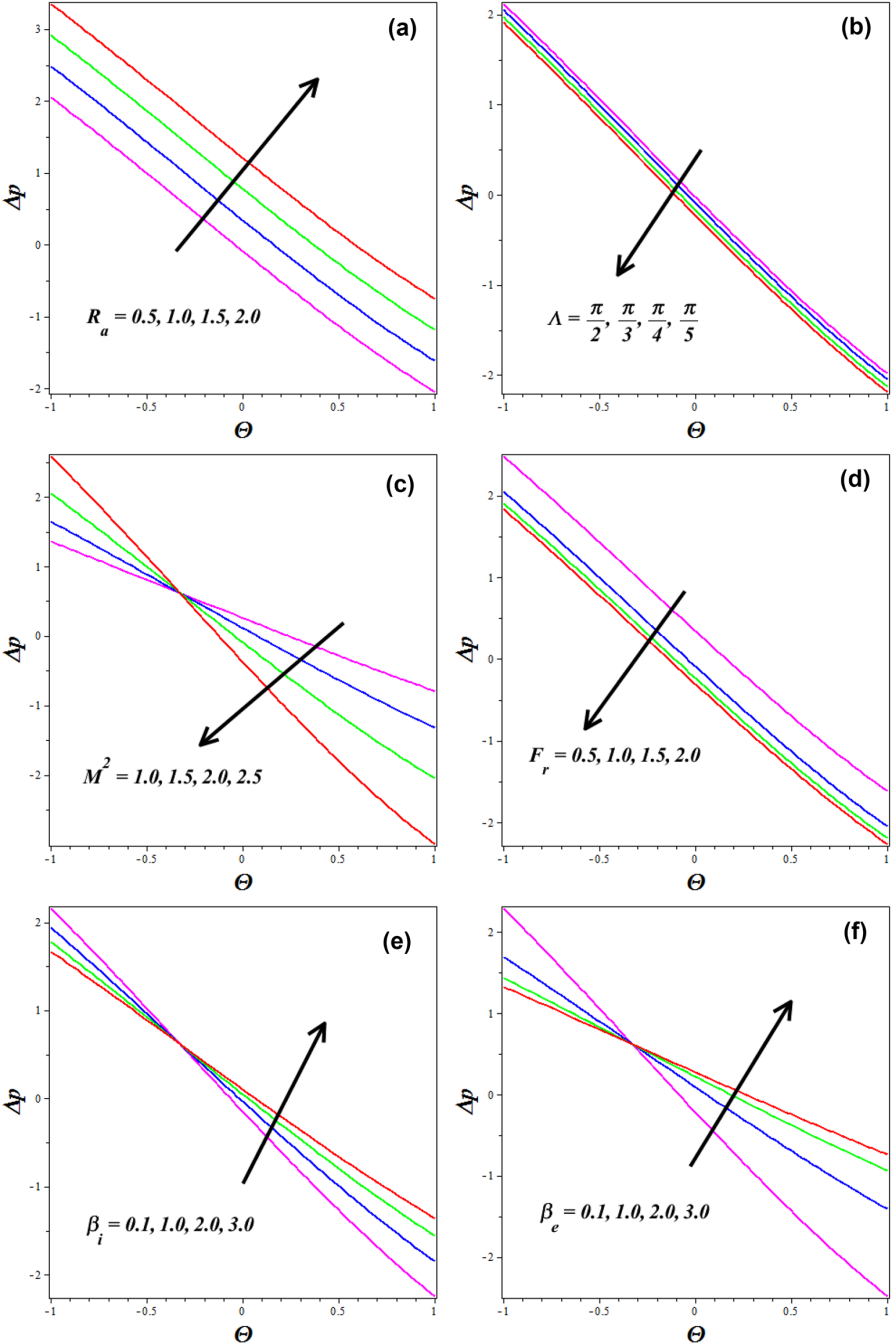
Influences of different parameters on Pressure gradient $\left( {\Delta p} \right)$.

A analytical integration was performed to analyze the pressure gradient $\left( {\Delta p} \right)$ per wavelength for Reynold's number $\left( {R_{a} } \right)$, channel inclination $\left( \Lambda \right)$, Hall parameter $\left( {\beta_{e} } \right)$, Hartmann number $\left( {M^{2} } \right)$, Froude number $\left( {F_{r} } \right)$, and ion-slip parameter $\left( {\beta_{i} } \right)$, as shown in Fig. 7a–f. Figure 7a shows that when the Reynolds numbers $\left( {R_{a} } \right)$ become better, the pressure rises even further. A fluid's Reynolds number $\left( {R_{a} } \right)$ is the ratio of its inertial and viscous forces. Because of this, the viscous forces tend to diminish and the velocity rises as increases. As a result, when the Reynolds number increases in peristaltic flow, more energy is dissipated due to turbulence, resulting in an increase in pressure rise. Turbulent flow patterns also cause more resistance to flow and, therefore, higher pressure gradients along the channel. This is why an increasing Reynolds number tends to lead to higher pressure rise in the peristaltic flow of fluids. Figure 7b displays how the pressure gradient profile and channel inclination are inversely connected. As the channel inclination increases, the pressure falls, as seen in the graph. Physically the altered flow patterns and increased frictional losses contribute to the overall pressure decrease. As the inclination parameter values rise, the gravitational influence becomes more dominant, leading to a gradual decrease in the pressure profile along the channel's length. Changes in the Hartmann number's $\left( {M^{2} } \right)$ effect on $\Delta p$ are seen in Fig. 7c. The peristaltic pumping zone shows that increasing values enhance pressure, whereas the retrograde pumping region shows the reverse trend, as seen in this graph. The increased Lorentz force in the channel caused by higher $\left( {M^{2} } \right)$ values strongly stimulates the electrically conducting nanofluid particles in the peristaltic pumping zone. As a result, the pressure in the pumping zone exhibits a falling behavior by raising $\left( {M^{2} } \right)$. Notably, the results of the study by Basha and Sivaraj^38^ are consistent with the impact of the magnetic field on pressure decrease. In Fig. 7d, the behavior of the Froude number $\left( {F_{r} } \right)$ on pressure gradient $\left( {\Delta p} \right)$ is portrayed. The graph shows that as the Froude number $\left( {F_{r} } \right)$ increases, the pressure falls. As the Froude number increases, the flow velocity becomes higher relative to the speed of gravity waves. In the supercritical flow regime, the flow experiences rapid changes in momentum and becomes more turbulent and unstable. This increased velocity leads to a substantial drop in pressure as the fluid's kinetic energy dominates over the potential energy due to gravity. Figure 7e, f describes how the pressure gradient $\left( {\Delta p} \right)$ gets influenced for the variations in the $\left( {\beta_{i} } \right)$ and $\left( {\beta_{e} } \right)$ respectively. It is observed that the pressure gradient $\left( {\Delta p} \right)$ augments by improving these both parameters $\left( {\beta_{i} } \right)$&$\left( {\beta_{e} } \right)$. This is because the presence of nanoparticles provides an additional barrier to fluid mobility. When the Hall parameter is relatively high, the Hall current becomes more dominant compared to the conduction current. This results in fluid particles experiencing additional deflections and acceleration perpendicular to the direction of the current flow. Consequently, the fluid flow becomes more organized, and there is an increased momentum transfer across the channel.

### Flow profiles

**Figure 8:**
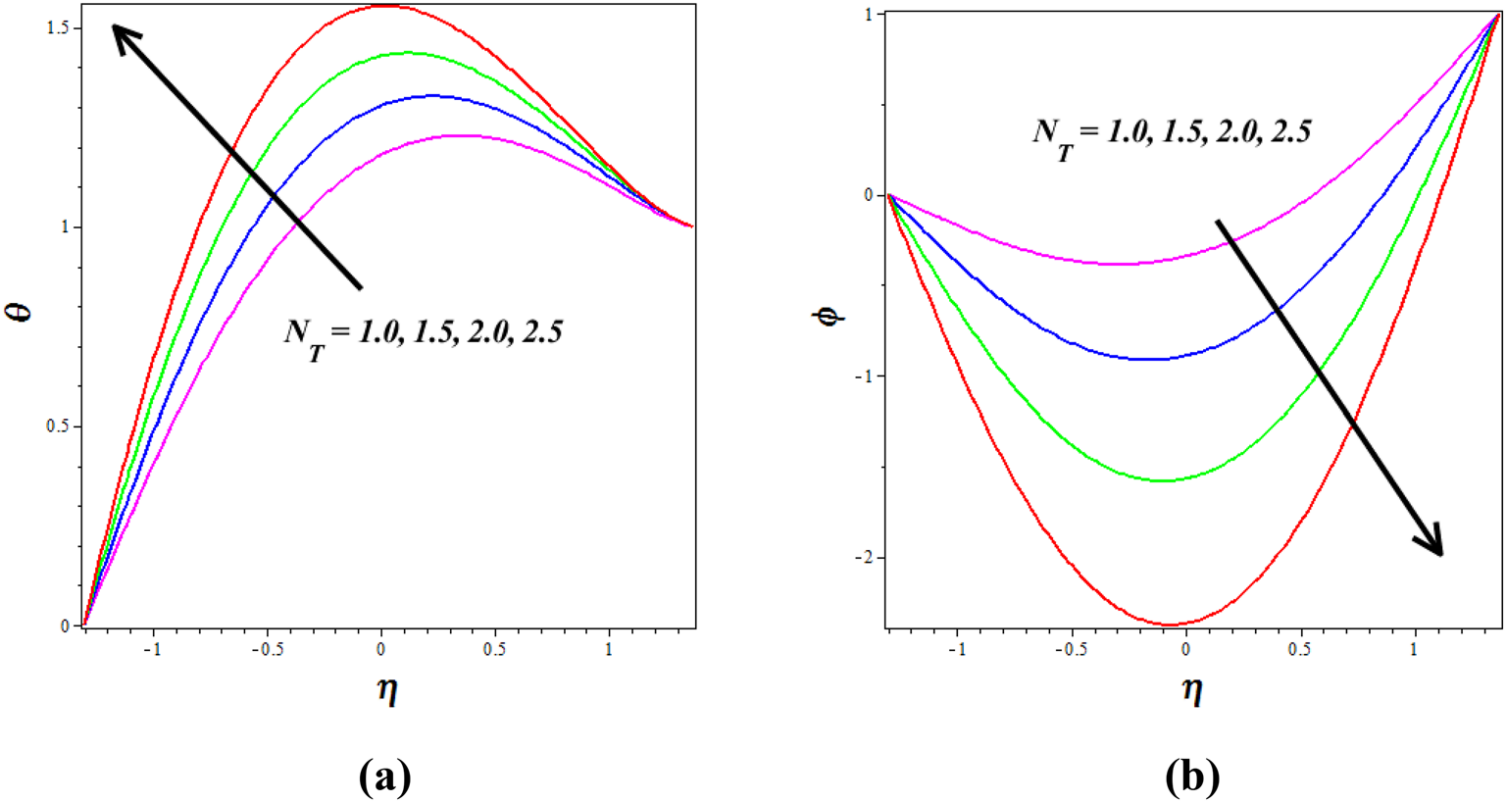
Influence of thermophoresis $\left( {N_{T} } \right)$ on (**a**) temperature $\left( \theta \right)$& (**b**) concentration $\left( \phi \right)$.

**Figure 9:**
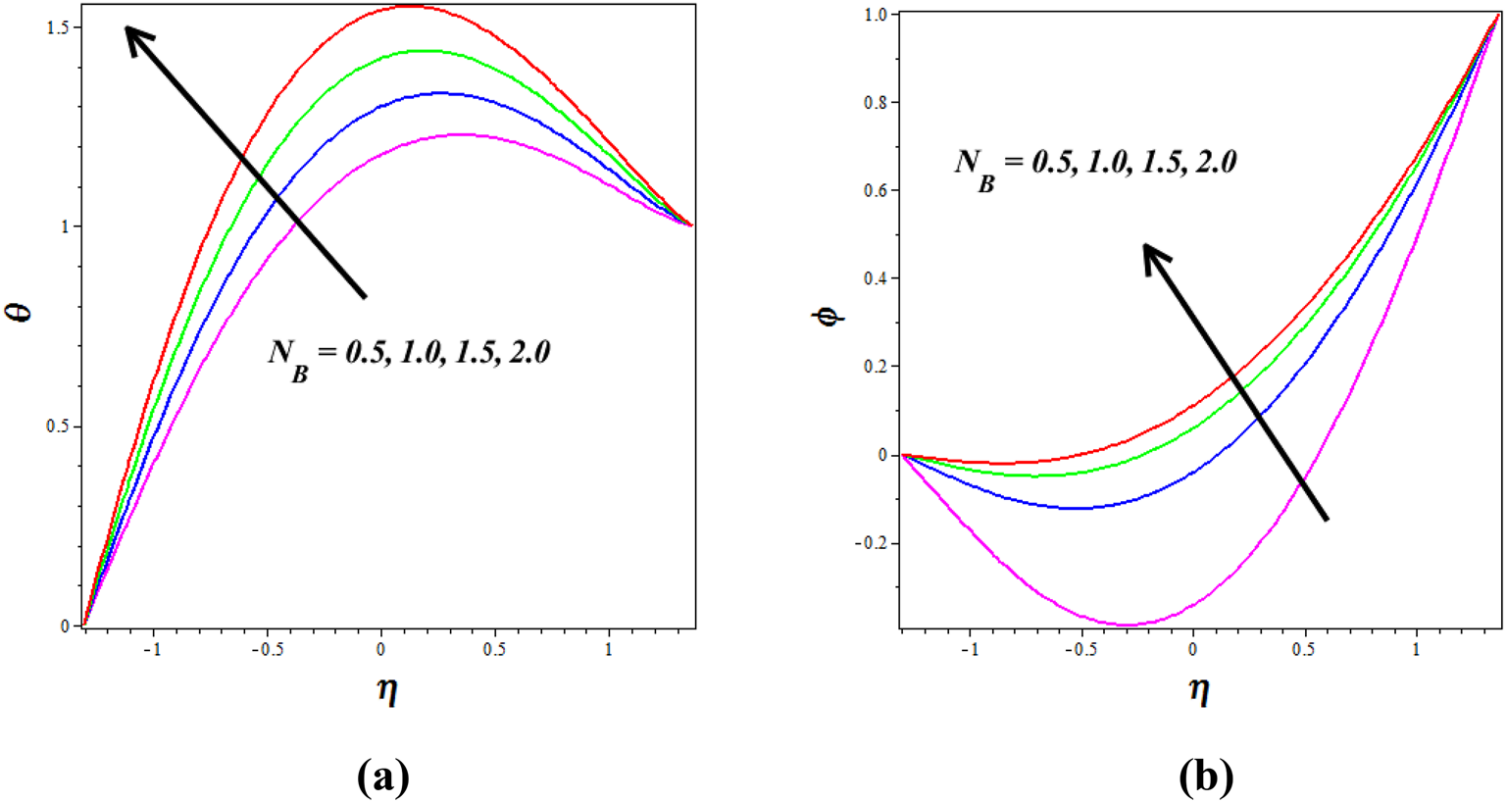
Influence of Brownian motion $\left( {N_{B} } \right)$ on (**a**) temperature $\left( \theta \right)$& (**b**) concentration $\left( \phi \right)$.

**Figure 10:**
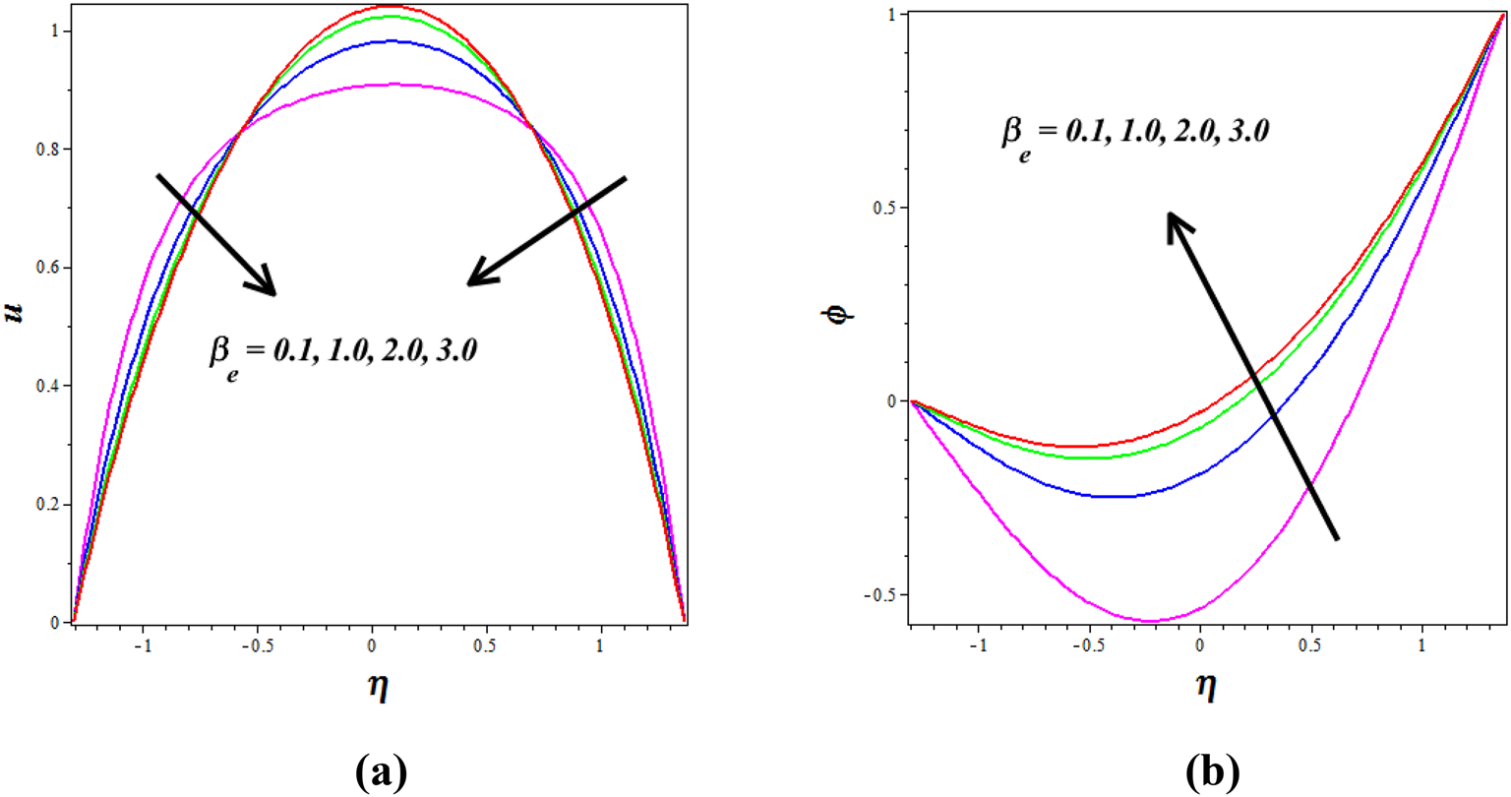
Influence of Hall parameter $\left( {\beta_{e} } \right)$ on (**a**) velocity $\left( u \right)$& (**b**) concentration $\left( \phi \right)$.

Figure 8a, b explores the changes of thermophoresis $\left( {N_{T} } \right)$ on nanofluid axial temperature $\left( \theta \right)$ and concentration $\left( \phi \right)$. It is noticed that thermophoresis improving for enhancing the $\left( \theta \right)$ and opposite nature is observed to $\left( \phi \right)$. The thermophoresis phenomenon describes the variations in solid nanoparticle mobility brought on by variations in temperature distribution. As thermophoresis values increase, solid nanoparticles start to travel quickly from hot to cold walls, accelerating the thermal distribution in the nanofluid and raising temperature in the process. The mass transport of nanoparticles is significantly distorted by this behavior in peristaltic transport, which lowers the concentration of nanoparticles. The phenomenon of thermophoresis is of significant importance in the field, particularly in the treatment of cancer where nanoparticles with high atomic numbers are utilized (Elmaboud et al.^33^,). Figure 9a, b describes how the $\left( \theta \right)$ and $\left( \phi \right)$ profiles gets influenced for the variations in Brownian motion $\left( {N_{B} } \right)$. As $\left( {N_{B} } \right)$ increases, nanoparticles move freely and irregularly in the blood nanofluid, which raises $\left( \theta \right)$ and the same nature is observed to $\left( \phi \right)$. The consequence of the Hall parameter $\left( {\beta_{e} } \right)$ on velocity $\left( u \right)$ and concentration $\left( \phi \right)$ is represented in Fig. 10a, b. It is observed that the velocity $\left( u \right)$ decrease by improving the Hall parameter $\left( {\beta_{e} } \right)$ and opposite nature is noticed in concentration $\left( \phi \right)$ profile. As the Hall parameter increases, the Hall current becomes more dominant compared to the conduction current. The Hall effect causes fluid particles to experience additional deflections and motion perpendicular to the direction of the electric current and magnetic field. This leads to increased flow velocities in the direction perpendicular to the flow. The amplified flow velocities in the transverse direction result in the stretching and elongation of the fluid flow, leading to a higher velocity profile. The effective conductivity is shown to decrease with increasing values of $\left( {\beta_{e} } \right)$, which also results in a reduction in the magnetic damping force and a corresponding decline in velocity.

**Figure 11:**
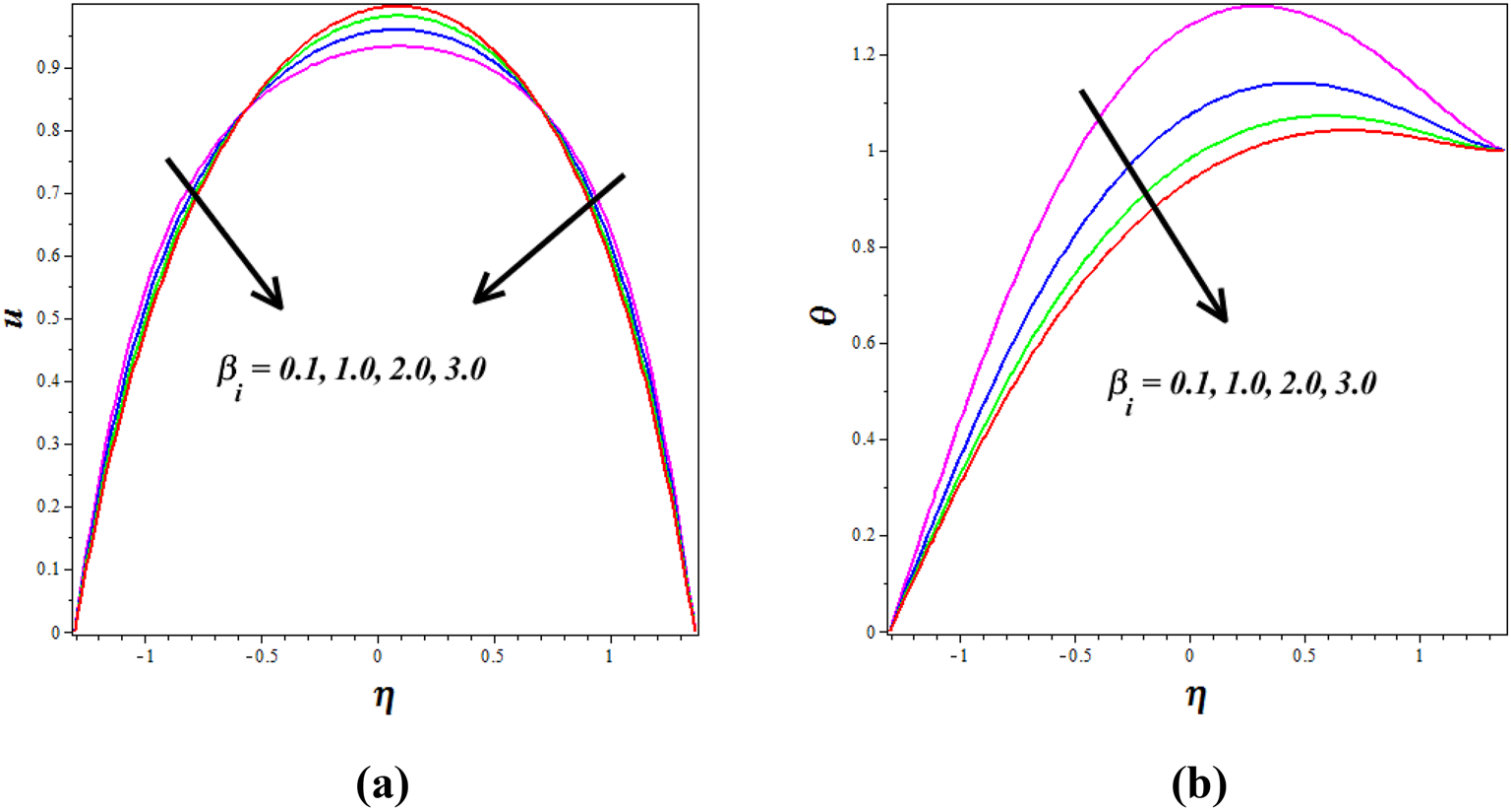
Influence of ion-slip parameter $\left( {\beta_{i} } \right)$ on (**a**) velocity $\left( u \right)$& (**b**) temperature $\left( \theta \right)$.

Figure 11a, b explores the changes of ion-slip $\left( {\beta_{i} } \right)$ on nanofluid axial axial $\left( u \right)$ and $\left( \theta \right)$. Enhancing ion-slip results in a decline in both fluid velocity and fluid temperature. Nevertheless, the central region of the channel exhibits a rising trend in fluid velocity. It is of significance to emphasize that the impact of the $\beta_{i}$ on temperature corresponds with the findings elucidated by Hayat et al.^33^.

**Figure 12:**
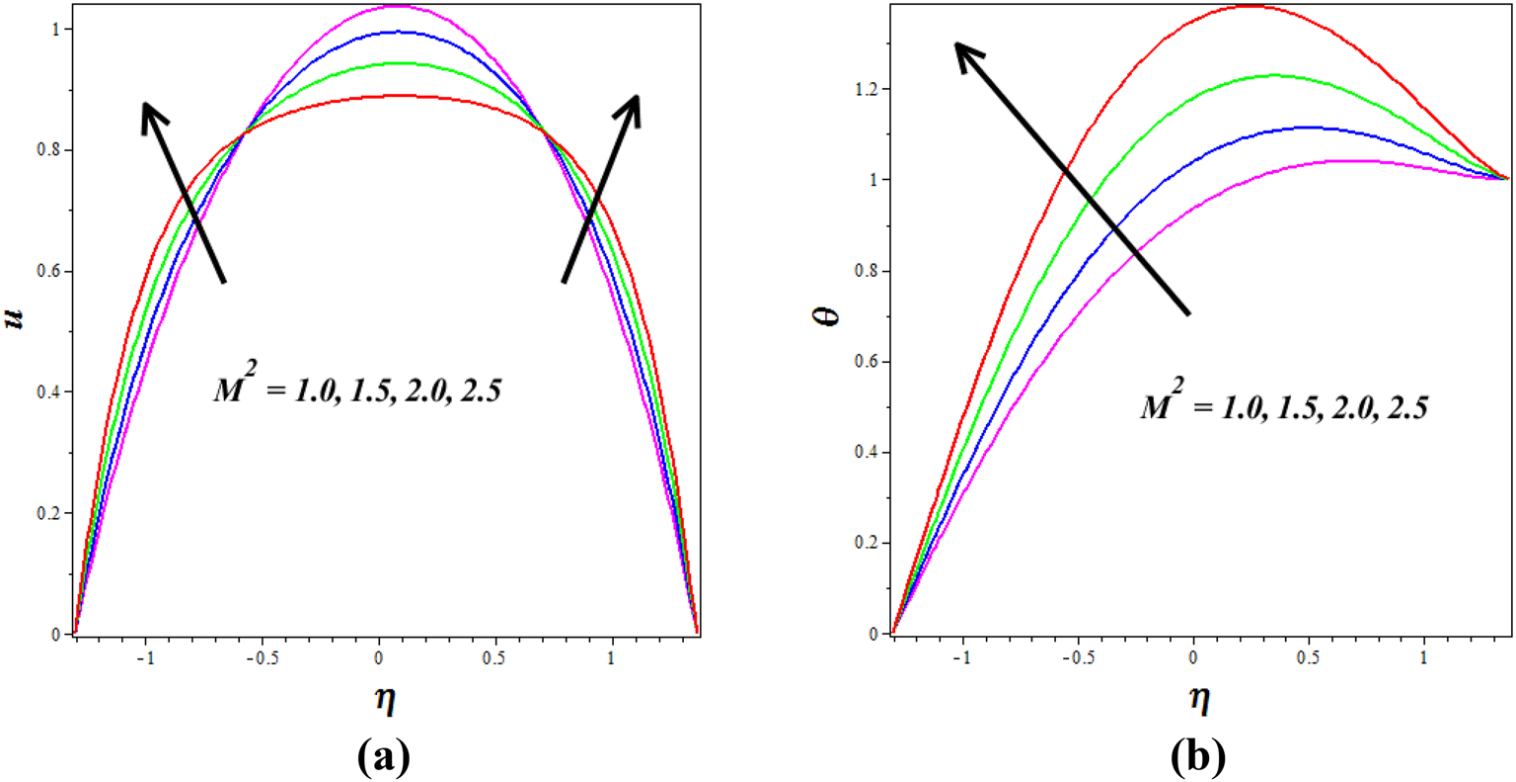
Influence of Hartmann number $\left( {M^{2} } \right)$ on (**a**) velocity $\left( u \right)$& (**b**) temperature $\left( \theta \right)$.

**Figure 13:**
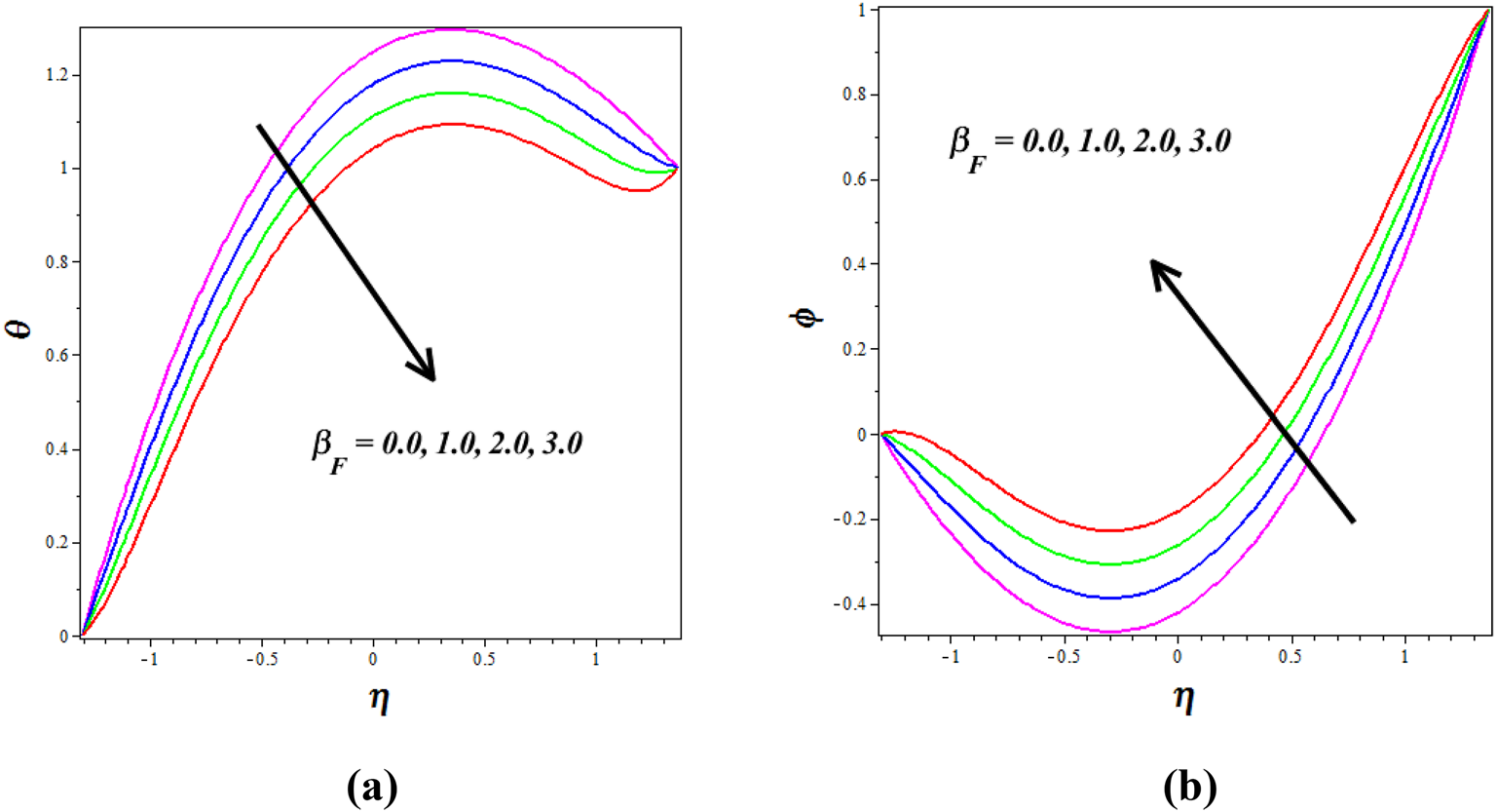
Influence of Sutterby fluid $\left( {\beta_{F} } \right)$ on (**a**) temperature $\left( \theta \right)$& (**b**) concentration $\left( \phi \right)$.

**Figure 14:**
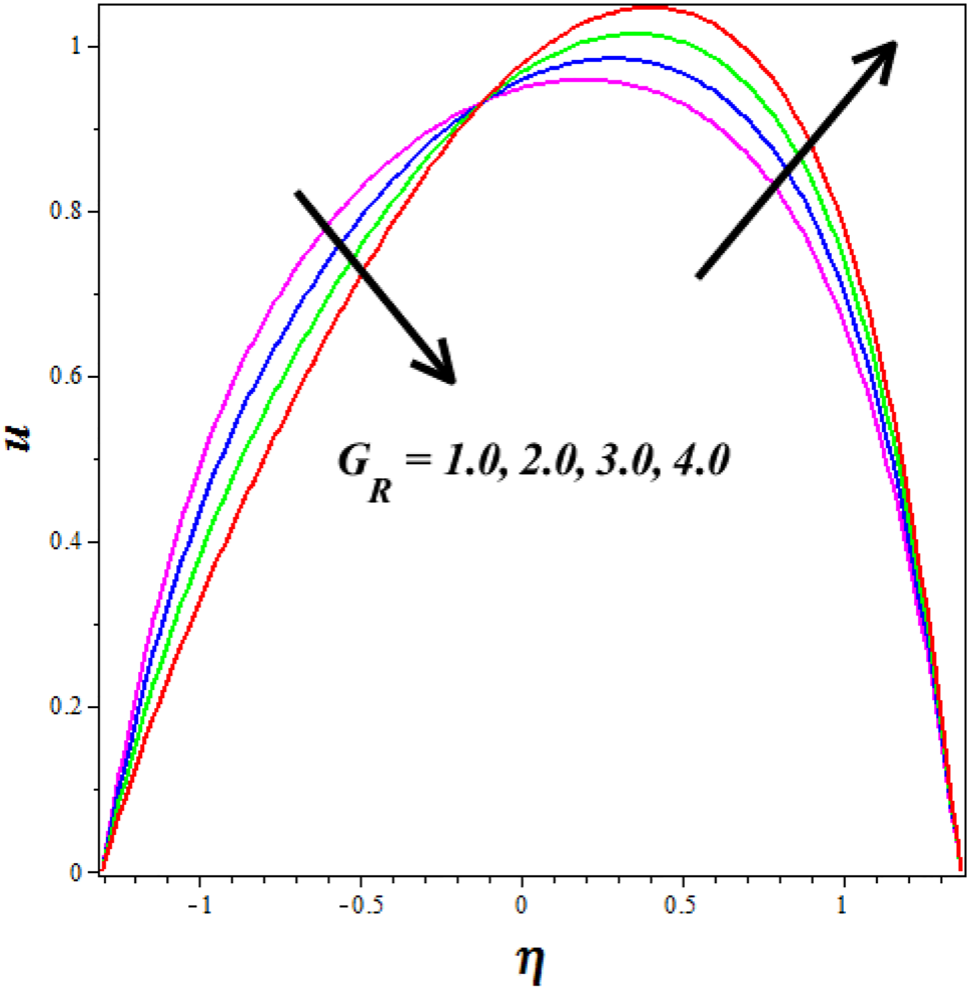
Influence of Grashof number $\left( {G_{R} } \right)$ on velocity $\left( u \right)$.

Figure 12a, b explores the changes of $\left( {M^{2} } \right)$ on nanofluid axial $\left( u \right)$ and $\left( \theta \right)$ silhouettes. It is noticed that $\left( {M^{2} } \right)$ improving for enhancing the $\left( u \right)$ and $\left( \theta \right)$ respectively. According to this graph, the $\left( u \right)$ and $\left( \theta \right)$ is improved by rising $\left( {M^{2} } \right)$ values in the peristaltic pumping zone whereas the retrograde pumping region exhibits the opposite tendency. The increased Lorentz force in the channel caused by higher $\left( {M^{2} } \right)$ values strongly stimulates the electrically conducting nanofluid particles in the peristaltic pumping zone. As a result, the $\left( u \right)$ and $\left( \theta \right)$ in the pumping zone exhibits a enlargements behavior by raising $\left( {M^{2} } \right)$. Figure 13a, b explores the changes of Sutterby fluid parameter $\left( {\beta_{F} } \right)$ on nanofluid $\left( \theta \right)$ and $\left( \phi \right)$ profiles. It is observed that the $\left( \theta \right)$ increase by improving the fluid parameter $\left( {\beta_{F} } \right)$ and opposite nature is noticed in concentration $\left( \phi \right)$ profile. Physically the Sutterby fluid parameter is high, the fluid's viscosity becomes more sensitive to changes in the flow rate and temperature. In regions of high flow velocity, the increased viscosity hinders the transfer of kinetic energy into thermal energy, resulting in a decrease in temperature. Conversely, in regions of low flow velocity, the higher viscosity leads to more significant resistance to flow, causing the fluid to concentrate and accumulate, resulting in an increase in the concentration profile. The consequence of the $\left( {G_{R} } \right)$ on $\left( u \right)$ is represented in Fig. 14. It is detected that levitation the values of $\left( {G_{R} } \right)$ causes the $\left( u \right)$ to grow. Physically the Grashof number increases, the buoyancy-driven convection becomes more pronounced, leading to stronger fluid motions and increased flow velocities. The fluid's velocity profile becomes more enhanced as it is driven by the buoyant forces, resulting in higher velocities near the heated walls and increased flow rates throughout the channel.

### Heat transfer profiles

**Figure 15:**
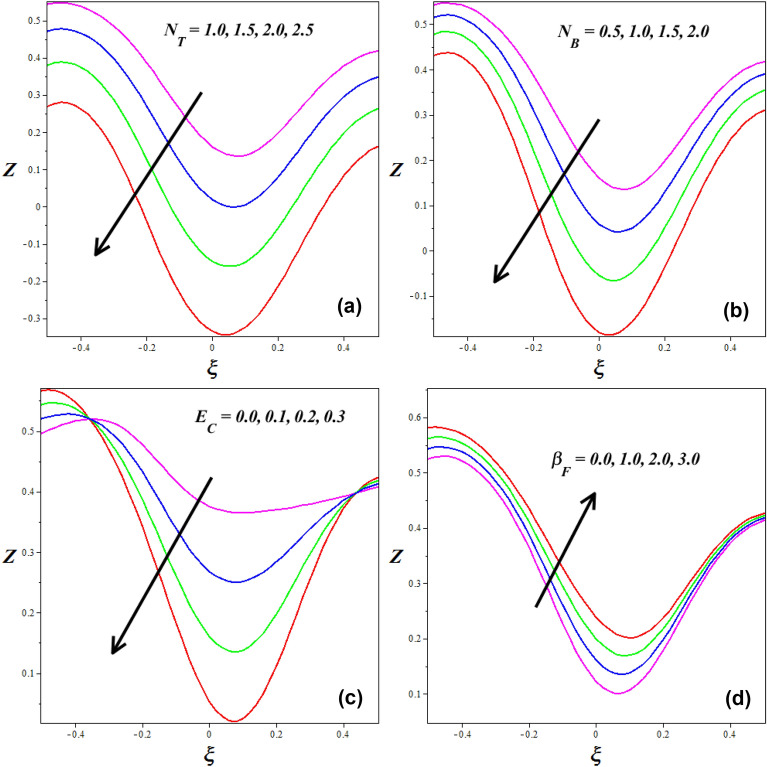
The effects of different factors on heat transfer.

Figure 15a–d is plotted for investigating the consequence of various active aspects in the heat transfer coefficient Z. Figure 15a, b describes how the heat transfer profiles gets influenced for the variations in $\left( {N_{B} } \right)$ and $\left( {N_{T} } \right)$. It is observed that heat transfer coefficient reducing for enhancing the $\left( {N_{B} } \right)$ and $\left( {N_{T} } \right)$ respectively. The thermophoresis phenomena explain changes in solid nanoparticle mobility caused by temperature distribution changes. As thermophoresis values rise, solid nanoparticles initiate to move gently from hotter to cooler walls, gradually thermal distribution in the nanofluid and lowering temperature. This tendency in peristaltic transport dramatically deviations the mass transfer of nanoparticles, lowering the concentration of nanoparticles. In Fig. 15c, the behavior of the Eckert number $\left( {E_{C} } \right)$ on heat transfer coefficient *Z* is portrayed. It is detected that heat transfer coefficient reducing for improving the values of $\left( {E_{C} } \right)$. Physically the Eckert number is high; the fluid's kinetic energy is more effective at dissipating thermal energy away from the heated surface. This enhanced kinetic energy carries away the heat more efficiently, reducing the temperature gradient near the solid surface. As a result, the thermal boundary layer thickness decreases, leading to a decrease in the heat transfer coefficient. Figure 15d shows the behavior of $\left( {\beta_{F} } \right)$ on heat transfer coefficient *Z* is portrayed. It is identified that heat transfer coefficient improving for improving the values of $\left( {\beta_{F} } \right)$.

### Isothermal profiles

**Figure 16:**
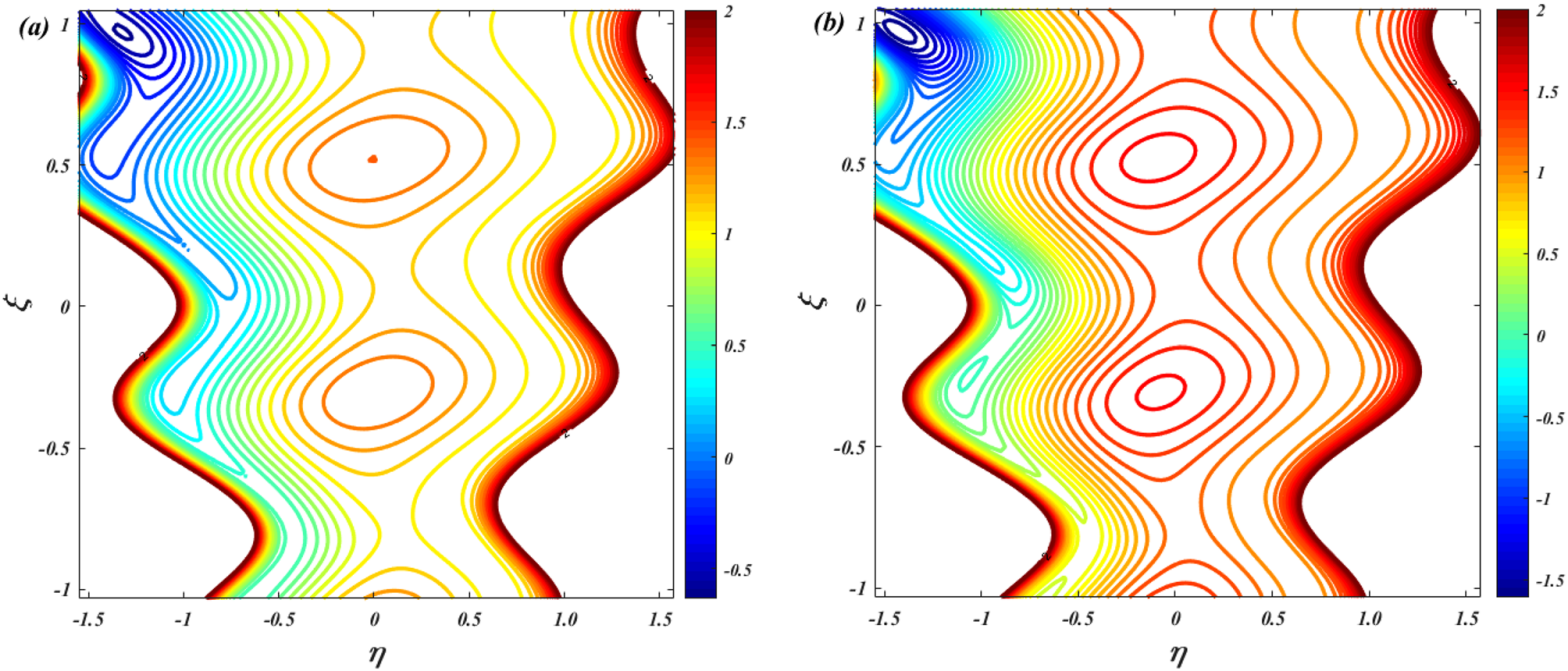
Isothermal distribution for thermophoresis $\left( {N_{T} = 1\& \,3} \right)$.

**Figure 17:**
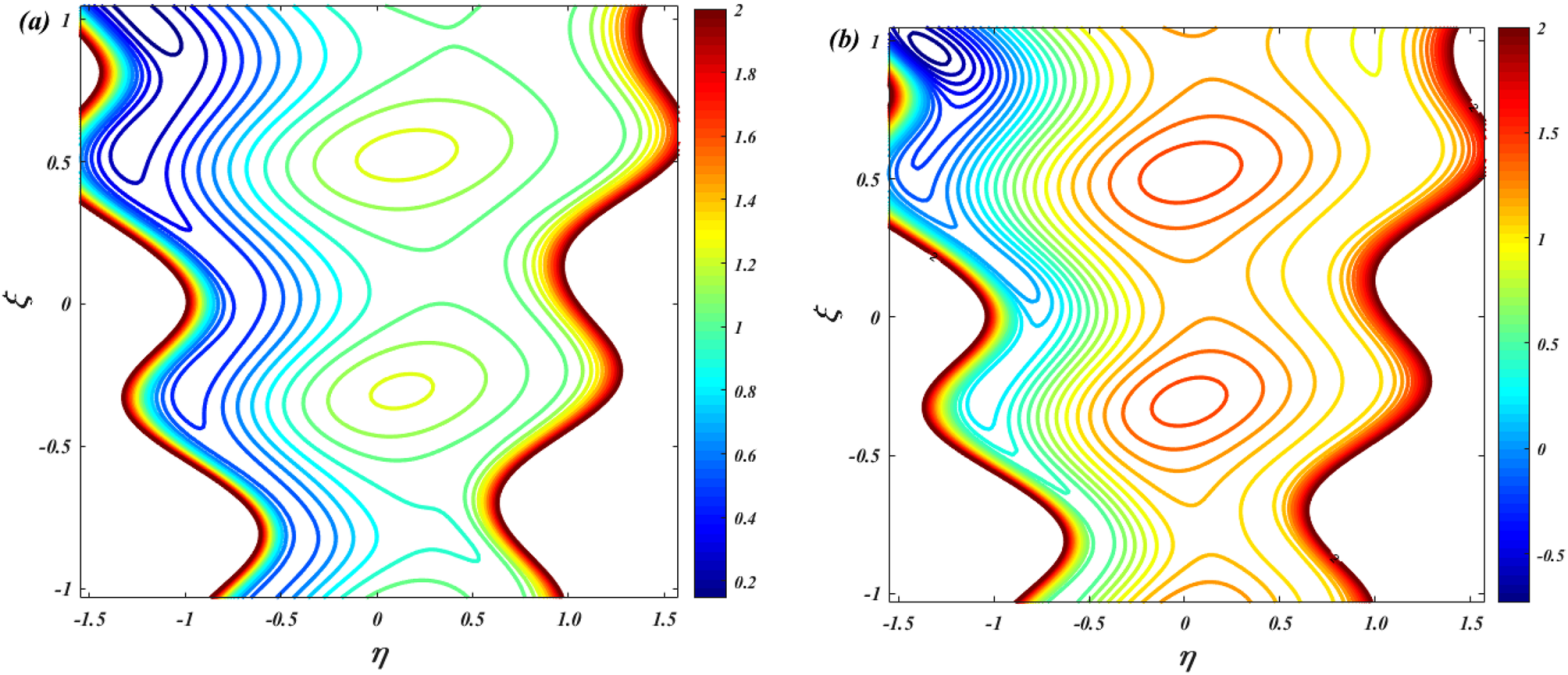
Isothermal distribution for Brownian motion $\left( {N_{B} = 1\& \,3} \right)$.

**Figure 18:**
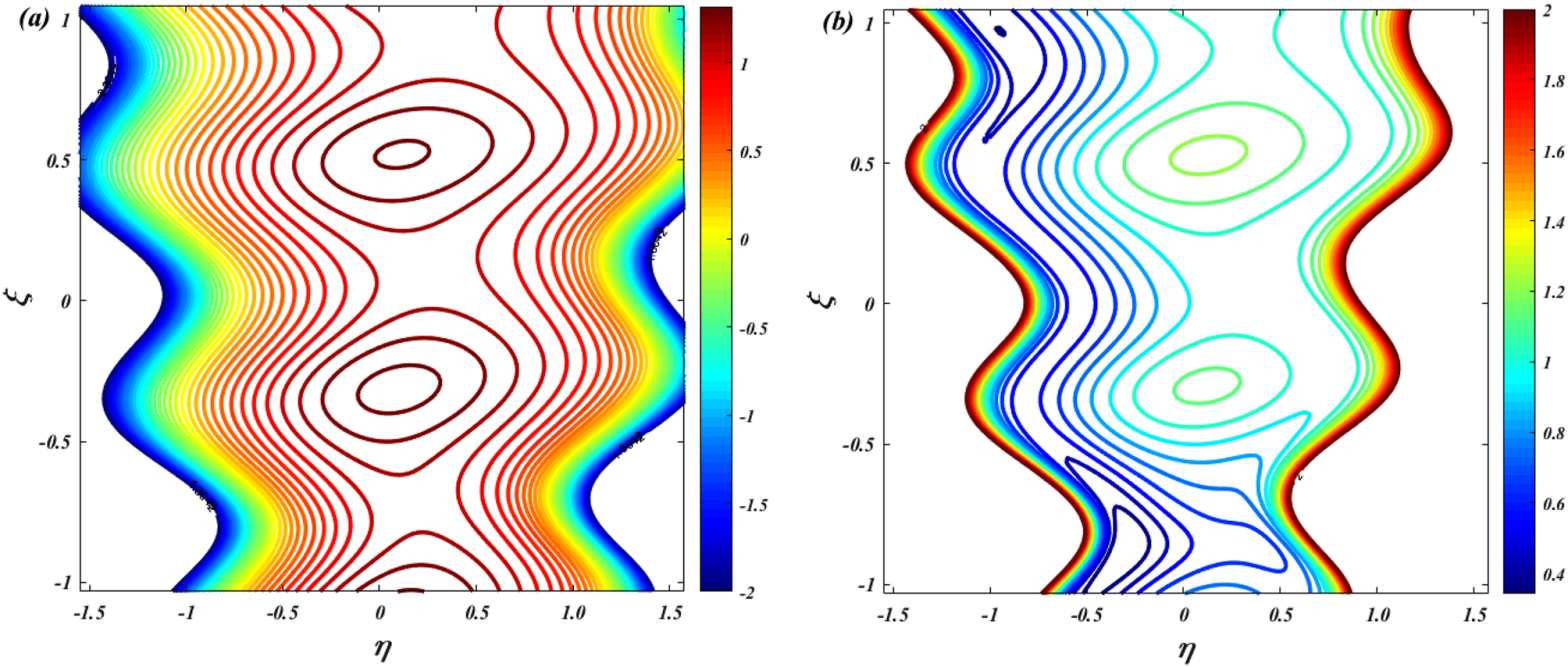
Isothermal distribution for Sutterby fluid parameter $\left( {\beta_{F} = 0\& \,3} \right)$.

In Fig. 16, the effects of the thermophoresis parameter on the isothermal lines are depicted. More heat is transported to the left when the thermophoresis parameter is increased, as seen by this graph. As the thermophoresis parameter increases, the temperature gradient becomes more pronounced. Consequently, the thermophoretic effect induces particles to move from regions of higher temperature (toward the heated right wall) to regions of lower temperature (toward the cooler left wall). This movement of particles carries the heat along with them, leading to the transport of heat toward the left wall. Figure 17 illustrates how the Brownian motion affects the isothermal lines of a system. This graphic shows that as the Brownian motion is raised, the rate of heat transfer increases and more heat is transferred to the left side. The increased Brownian motion facilitates the movement of particles toward both the heated and cooler regions, leading to a more even distribution of particles and heat in the fluid. As a result, more heat is transferred to the left side as particles carry thermal energy from the heated regions and disperse it throughout the fluid, mitigating temperature gradients. Figure 18 describes how the isothermal lines gets influenced for the variations in fluid parameter $\left( {\beta_{F} } \right)$. The heat transfer is decreasing when enhancing the fluid parameter and heat is transformed to right and left side of the walls. The increased non-Newtonian effects in the Sutterby fluid result in higher viscosity and shear-thinning behavior. Higher viscosity hinders the fluid's ability to transfer heat efficiently, reducing the heat transfer rate. Additionally, shear-thinning behavior causes a decrease in fluid momentum near the walls, leading to reduced heat transfer in these regions.

### Streamline

**Figure 19:**
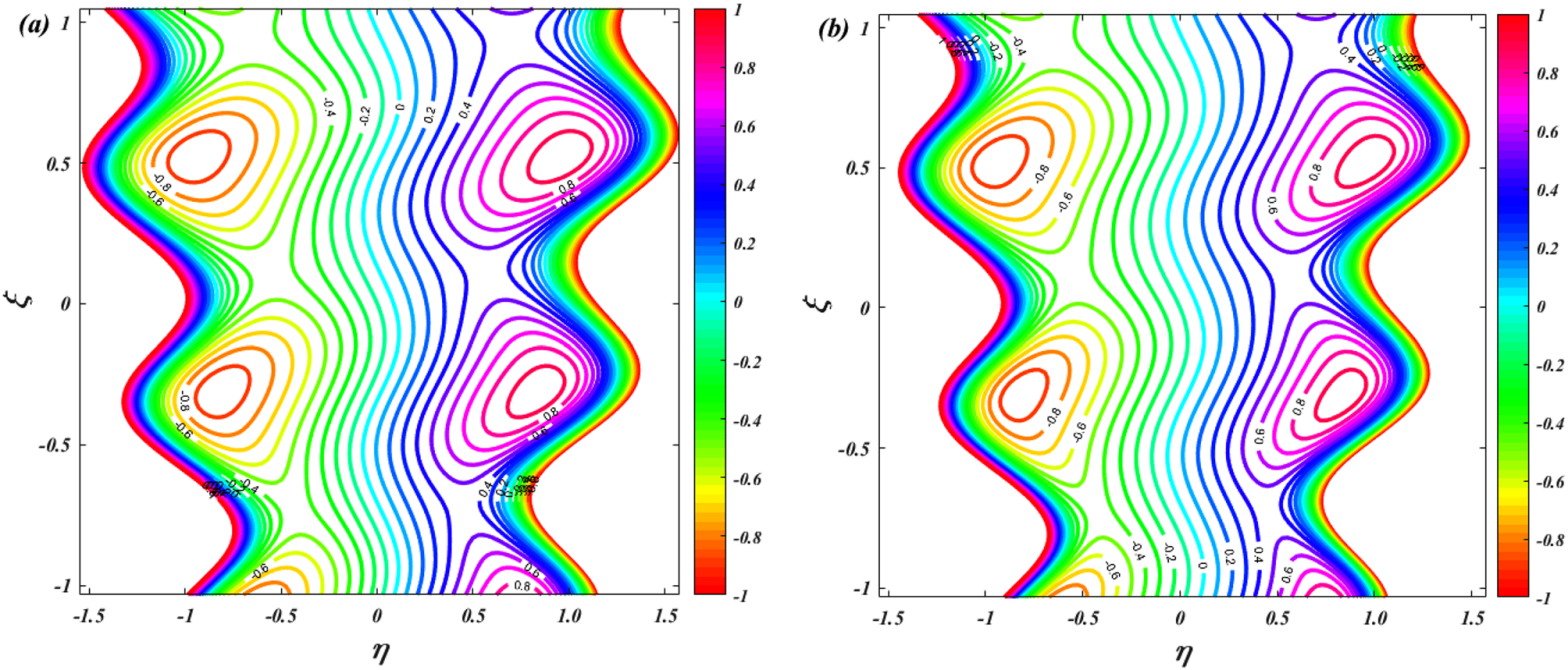
Streamline distribution for ion-slip parameter $\left( {\beta_{i} = 1\& \,3} \right)$.

**Figure 20:**
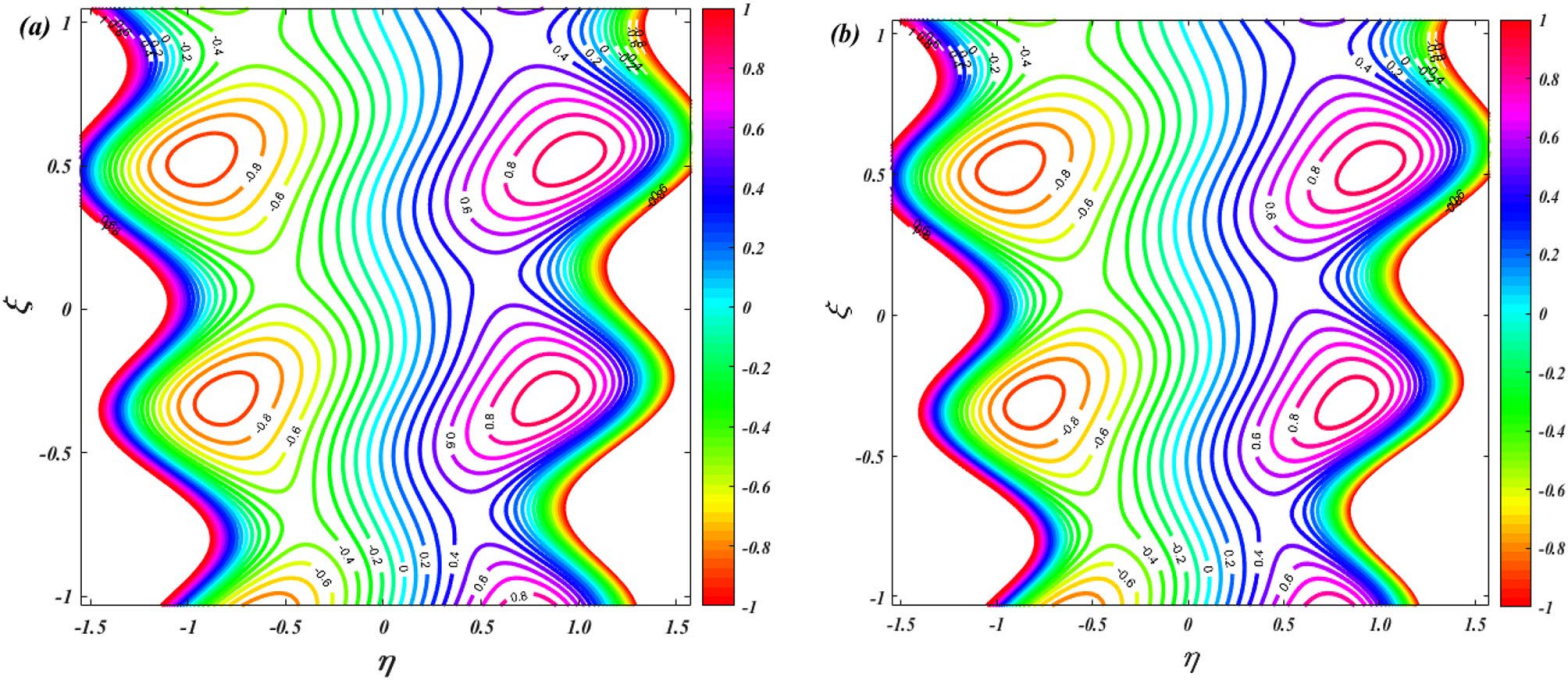
Streamline distribution for Sutterby fluid parameter $\left( {\beta_{F} = 0\& \,3} \right)$.

**Figure 21:**
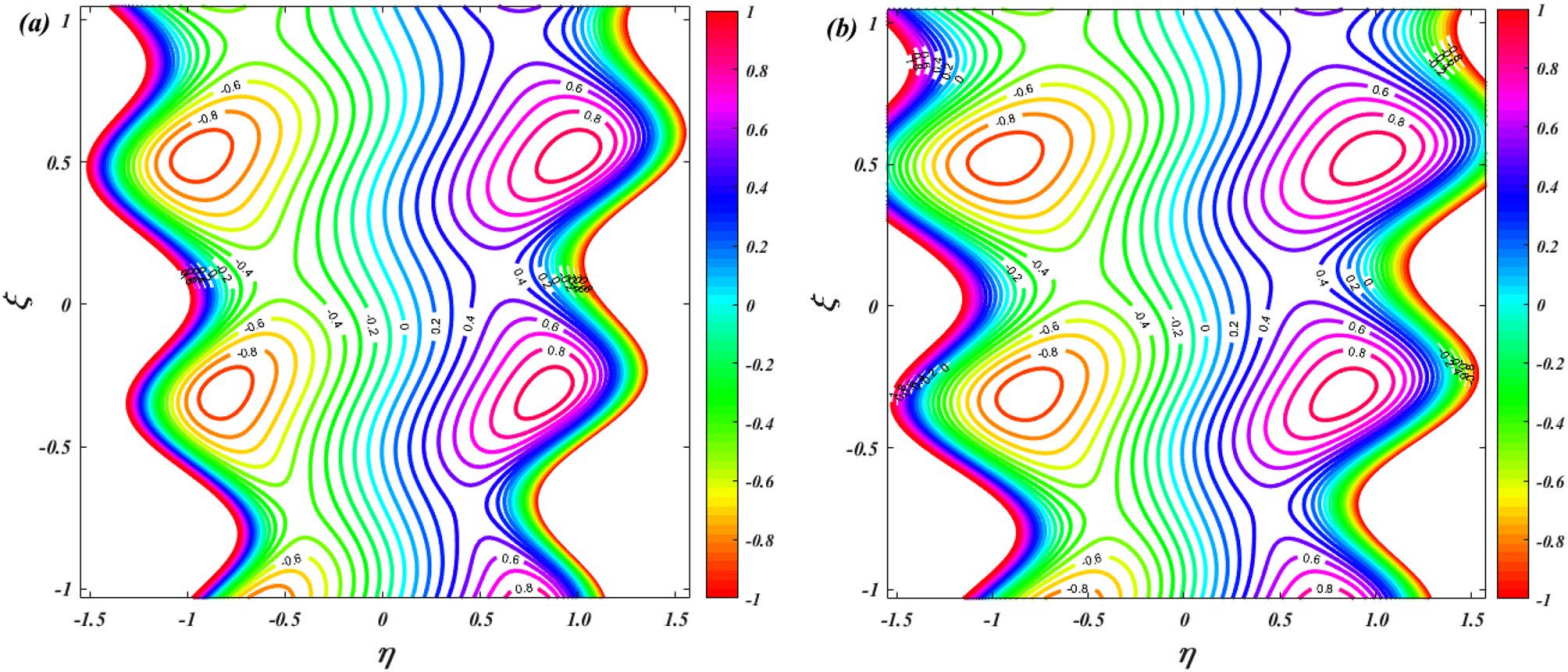
Streamline distribution for Hall parameter $\left( {\beta_{e} = 0\& \,3} \right)$.

Figure 19 is depicts to examine the characteristics of ion-slip parameter on streamlines. When increasing the values of ion-slip parameter from $\beta_{i} = 1$ to $\beta_{i} = 3$ the fluid flow in the channel is slightly decreasing. As the ion-slip parameter increases, the ion drag effect becomes more pronounced, resulting in a slight decrease in fluid momentum near the channel walls. This reduced momentum leads to a small decrease in the fluid flow velocity and can cause a slight decrease in the overall flow rate along the channel. Figure 20 is represents to study the characteristics of sutterby fluid parameter on streamlines. When increasing the values of sutterby fluid parameter from $\beta_{F} = 0$ to $\beta_{F} = 3$ there is no significance influence on the streamlines. Figure 21 depicts the influence of Hall parameter on streamlines. This graph shows that raising Hall parameter causes the trapping bolus density to increases.

## Conclusion

The investigation aimed to show the effects of hall current and ion slip on the fluid flow, heat transfer, and mass transfer characteristics of peristaltic bio-Sutterby blood nanofluid flow in an inclined tapered channel. The impact of thermophoresis and Brownian motion on nanoparticles was shown using a non-homogeneous nanofluid model. In order to transform dimensional partial differential equations into dimensionless form using non-similar variables, the Homotopy perturbation technique is used. Two-dimensional streamlines and isotherm graphs are used to predict the major parameters of velocity, pressure drop, and temperature. The main results of the model are as follows.

1. The artificial neural network model does not require linearization, is fast convergent, and has a reduced processing cost.
2. In the isothermal profiles, the rate of heat transfer increases due to Brownian motion being increased.
3. The temperature profile $\left( \theta \right)$ increase by improving the fluid parameter $\left( {\beta_{F} } \right)$ and opposite nature is noticed in concentration $\left( \phi \right)$ profile.
4. Higher values of the Reynolds numbers $\left( {R_{a} } \right)$ increase the pressure gradient $\left( {\Delta p} \right)$.
5. Thermophoresis parameter improving for enhancing the temperature profile $\left( \theta \right)$ and opposite nature is observed to concentration profile $\left( \phi \right)$.
6. The heat transfer coefficient reducing for improving the values of Eckert number.
7. The mean square error in target values is 0.000000209144 by the artificial neural network approach.

Future research based on the presented model of hydromagnetic peristaltic transport of Sutterby nanofluid in an inclined tapered channel can explore several key areas to advance the understanding and applications in diverse fields. The experimental validation of the model's predictions could enhance its reliability and applicability in real-world scenarios. Additionally, conducting sensitivity analyses to identify the most influential parameters and optimization studies to optimize flow characteristics and heat transfer efficiency could lead to practical advancements in engineering applications. Extending the investigation to more complex geometries and multi-dimensional systems, as well as exploring the behavior of other non-Newtonian nanofluids, would broaden the scope of the study. In conclusion, future research should aim to enhance predictive capabilities, explore various non-Newtonian fluid behaviors, and address complex fluid dynamics problems for practical implications in diverse industrial and biomedical fields.

## Data Availability

The datasets used and/or analysed during the current study available from the corresponding author on reasonable request.

## Abbreviations

$$p^{ * }$$: Dimensional pressure
$\tau^{ * }_{{\xi^{ * } \xi^{ * } }} ,\tau^{ * }_{{\xi^{ * } \eta^{ * } }} ,\tau^{ * }_{{\eta^{ * } \eta^{ * } }}$: Extra stress tensors
$$t^{ * }$$: Dimensional time
$K_{1}$: Dimensional chemical reaction
$T_{0}$: Right wall temperature
$T_{1}$: Left wall temperature
$$M^{2}$$: Hartmann number
$$G_{r}$$: Grashof number
$C_{w}$: Nanoparticles concentration at the wall
$$Ra$$: Reynold’s number
*Ec*: Eckert number
$C_{1}$: Left wall concentrations
$C_{0}$: Right wall concentrations
$K_{R}$: Chemical reaction parameter
$\lambda$: Mixed convection parameter
$$c_{p}$$: Specific heat
$v^{ * }$: Velocity component along $\eta^{ * }$-direction
$\phi$: Dimensionless nanoparticles concentration
$$F_{r}$$: Froude number
$\gamma_{1}$: Joule heating parameter
$$We$$: Weissenberg number
$$\rho$$: Density
$$\theta$$: Dimensionless temperature
$$\mu$$: Dynamic viscosity
$$\upsilon$$: Kinematic viscosity
$$\sigma$$: Electrical conductivity
*f*: Fluid
*N*_*B*_: Brownian motion parameter
$$k$$: Thermal conductivity
*N*_*T*_: Thermophoresis parameter
*Pr*: Prandtl number
*Sc*: Schmidt number
$D_{B}$: Brownian diffusion coefficient
*u**: Velocity component along* χ**-direction
$D_{B}$: Thermophoresis diffusion coefficient
