# Publisher Correction: Peristaltic transport of Sutterby nanofluid flow in an inclined tapered channel with an artificial neural network model and biomedical engineering application

**DOI:** 10.1038/s41598-024-53423-3

**Published:** 2024-02-05

**Authors:** P. Chinnasamy, R. Sivajothi, S. Sathish, Mohamed Abbas, V. Jeyakrishnan, Rajat Goel, Mohammed S. Alqahtani, K. Loganathan

**Affiliations:** 1 Department of Computer Science and Engineering, MLR Institute of Technology, Hyderabad, Telangana India; 2 Department of Management, R L Institute of Management Studies (A Unit of Subbalakshmi Lakshmipathy College of Science), Madurai, Tamil Nadu India; 3 Department of Mathematics, School of Science, National Institute of Technology, Tadepalligudem, Andhra Pradesh India; 4 Electrical Engineering Department, College of Engineering, King Khalid University, 61421 Abha, Saudi Arabia; 5 Department of Computer Science and Engineering, Manipal University Jaipur, Jaipur, Rajasthan 303007 India; 6 Radiological Sciences Department, College of Applied Medical Sciences, King Khalid University, 61421 Abha, Saudi Arabia; 7 BioImaging Unit, Space Research Centre, Michael Atiyah Building, University of Leicester, Leicester, LE1 7RH UK; 8 Department of Mathematics and Statistics, Manipal University Jaipur, Jaipur, Rajasthan 303007 India

Correction to: *Scientific Reports* 10.1038/s41598-023-49480-9, published online 04 January 2024

In the original version of this Article Rajat Goel was incorrectly affiliated with ‘Radiological Sciences Department, College of Applied Medical Sciences, King Khalid University, 61421 Abha, Saudi Arabia’. The correct affiliation is listed below.

Department of Computer Science and Engineering, Manipal University Jaipur, Jaipur, Rajasthan 303007, India.

The original Article has been corrected.

